# Cost effectiveness of preemptive school closures to mitigate pandemic influenza outbreaks of differing severity in the United States

**DOI:** 10.1186/s12889-023-17469-8

**Published:** 2024-01-17

**Authors:** Lori R. Dauelsberg, Brian Maskery, Heesoo Joo, Timothy C. Germann, Sara Y. Del Valle, Amra Uzicanin

**Affiliations:** 1 Analytics, Intelligence and Technology Division, Los Alamos National Laboratory, PO Box 1663, Los Alamos, NM 87545 USA; 2 Division of Global Migration and Quarantine, National Center for Emerging and Zoonotic Infectious Diseases, Centers for Disease Control and Prevention, 1600 Clifton Road NE, MS H16-4, Atlanta, GA 30329 USA; 3 Theoretical Division, Los Alamos National Laboratory, Los Alamos, USA

**Keywords:** Community mitigation, Economics, Influenza, Nonpharmaceutical interventions, Pandemic, Preemptive school closures, Societal costs

## Abstract

**Background:**

Nonpharmaceutical interventions (NPIs) may be considered as part of national pandemic preparedness as a first line defense against influenza pandemics. Preemptive school closures (PSCs) are an NPI reserved for severe pandemics and are highly effective in slowing influenza spread but have unintended consequences.

**Methods:**

We used results of simulated PSC impacts for a 1957-like pandemic (i.e., an influenza pandemic with a high case fatality rate) to estimate population health impacts and quantify PSC costs at the national level using three geographical scales, four closure durations, and three dismissal decision criteria (i.e., the number of cases detected to trigger closures). At the Chicago regional level, we also used results from simulated 1957-like, 1968-like, and 2009-like pandemics. Our net estimated economic impacts resulted from educational productivity costs plus loss of income associated with providing childcare during closures after netting out productivity gains from averted influenza illness based on the number of cases and deaths for each mitigation strategy.

**Results:**

For the 1957-like, national-level model, estimated net PSC costs and averted cases ranged from $7.5 billion (2016 USD) averting 14.5 million cases for two-week, community-level closures to $97 billion averting 47 million cases for 12-week, county-level closures. We found that 2-week school-by-school PSCs had the lowest cost per discounted life-year gained compared to county-wide or school district–wide closures for both the national and Chicago regional-level analyses of all pandemics. The feasibility of spatiotemporally precise triggering is questionable for most locales. Theoretically, this would be an attractive early option to allow more time to assess transmissibility and severity of a novel influenza virus. However, we also found that county-wide PSCs of longer durations (8 to 12 weeks) could avert the most cases (31–47 million) and deaths (105,000–156,000); however, the net cost would be considerably greater ($88-$103 billion net of averted illness costs) for the national-level, 1957-like analysis.

**Conclusions:**

We found that the net costs per death averted ($180,000-$4.2 million) for the national-level, 1957-like scenarios were generally less than the range of values recommended for regulatory impact analyses ($4.6 to 15.0 million). This suggests that the economic benefits of national-level PSC strategies could exceed the costs of these interventions during future pandemics with highly transmissible strains with high case fatality rates. In contrast, the PSC outcomes for regional models of the 1968-like and 2009-like pandemics were less likely to be cost effective; more targeted and shorter duration closures would be recommended for these pandemics.

**Supplementary Information:**

The online version contains supplementary material available at 10.1186/s12889-023-17469-8.

## Background

Influenza pandemics occur after a novel, readily transmissible influenza virus emerges and spreads rapidly across the globe [1]. They are inherently unpredictable with regard to timing—over the past 100 years, only 4 have occurred—and vary greatly with regard to transmissibility and clinical severity [1].^1^ Community mitigation is part of the national pandemic response plan as the first line of defense for influenza pandemics until pandemic influenza vaccines are widely available [2]. Community mitigation refers to nonpharmaceutical interventions (NPIs), which are a set of actions that people and communities can take to slow the spread of disease [3]. Preemptive school closures (PSCs), a community NPI reserved for use in pandemics, are implemented before disease becomes widespread in schools and communities and may be recommended during severe, very severe, and extreme influenza pandemics to achieve one or more of the following specific public health objectives [3]:

- 1: “To gain time for an initial assessment of transmissibility and clinical severity of the pandemic virus in the very early stage of its circulation in humans (closures up to 2 weeks).”
- 2: “To slow down the spread of the pandemic virus in areas that are beginning to experience local outbreaks and thereby allow time for the local health care system to prepare additional resources for responding to increased demand for health care services (closures up to 6 weeks).”
- 3: “To allow time for pandemic vaccine production and distribution (closures up to 6 months” [3] – the duration is related with the presently anticipated influenza vaccine production and distribution timelines).

This recommendation is based on a 2012 statement by the U.S. Community Preventive Services Task Force (CPSTF) that recommended coordinated PSCs and dismissals during a severe influenza pandemic (a pandemic with high rates of severe illness such as that experienced in 1918) based on sufficient evidence of effectiveness in reducing or delaying the spread of infection and illness within communities [4]. However, the CPSTF found insufficient evidence to determine the balance of benefits and harms of preemptive, coordinated school dismissals in the event of an influenza pandemic of moderate or less severity because few studies provide comparative information relevant to an overall assessment of potential benefits and costs of school dismissals for pandemics without high rates of severe illness [4].

In this study, we present an economic evaluation from the societal perspective of PSCs implemented during a hypothetical influenza pandemic similar in magnitude to the influenza pandemic in 1957 at the national level as well as 1968-like and 2009-like pandemic scenarios for the Chicago region [5]. While the 1957 influenza pandemic was substantially less severe than that of 1918, a contemporary pandemic severity analysis shows it as the most impactful modern-time influenza pandemic with a relatively higher case fatality rate [6]. Given this constellation of factors, we chose to present the 1957-like pandemic severity scenario for the national-level economic evaluation here and include additional regional scenarios evaluated primarily in the Supplemental Information. Our analysis explores the national economic impacts for the scenario of greatest severity, for which coordinated PSCs are most likely to be relevant both at the state and at the national level. We also explore a larger set of assumptions and pandemic scenarios based on a set of regional “deep dive” epidemiologic model simulations for less severe pandemic scenarios. The goal of this study was to explore costs and cost effectiveness associated with PSCs as a countermeasure aimed to slow down or lessen the pandemic-associated morbidity and mortality, compared with the cost of a pandemic without this mitigation measure.

## Methods

To estimate the cost effectiveness of PSCs based on the 1957-like pandemic, we used the pertinent parameter assumptions and model-produced epidemiologic data from a previously reported national-level 1957-like pandemic simulation. The parameters carried over from that work into the present analysis are summarized in Table 1 and Supplemental Information Tables S1-S2 The results of the epidemiologic model included the total number of pandemic influenza cases averted, if the schools are closed, as well as the number of schools closed and the number of students affected (i.e., out of school). More details on the data inputs, model structure, assumptions and calibration of the simulation model and related limitations are included in this earlier publication [5]. The analysis presented here are an extension of that earlier work in that they provide additional context by estimating the costs of PSCs and conducting cost effectiveness analyses of the PSC strategies. We used these previously published results as input parameters in the present economic analysis to determine the total costs of illness, including hospitalizations, for averted cases and the costs to close schools.

**Table 1:** Parameter values and assumptions informing an cost effectiveness analysis of preemptive school closures to mitigate pandemic influenza outbreaks

Parameter Description	Base Value	Range	Source
**Epidemiologic Model Parameters **(Germann et al. 2019) [5]			
Population	National: 281 million people Regional (Chicago): 8.6 million people	NA	US Census, 2000
Influenza attack rate in the absence of intervention^a^	National and Regional (Chicago) B2 Model (1957 like) Children: 50%; Adults: 23%; Elderly: 11%; Overall: 28%	NA	[5–7]
	National and Regional (Chicago) B1 Model (1968 like) Children: 39%; Adults: 18%; Elderly: 8%; Overall: 22%		
	National and Regional (Chicago) A Model (2009 like) Children: 32%; Adults: 15%; Elderly: 7%; Overall: 18%		
R_0_^a^	National and Regional (Chicago) B2 Model (1957 like): 1.8	NA	[5–7]
	National and Regional (Chicago) B1 Model (1968 like): 1.5		
	National and Regional (Chicago) A Model (2009 like): 1.3		
Disease parameters^a^	Latent Period: 1.2 days; Incubation Period: 1.9 days; Infectious Period: 4.1 days; Mean Serial Interval: 3.95 days	NA	[5]
Case fatality rate^a^	National and Regional (Chicago) B2 Model (1957 like) 0–4: 0.05%; 5–18: 0.01%; 19–29: 0.09%; 30–64: 0.14%; 65 + : 3.81%	Flat rate of 0.1% across all age groups to base case	[5, 7, 8]
	National and Regional (Chicago) B1 Model (1968 like) 0–4: 0.01%; 5–18: 0.00%; 19–29: 0.03%; 30–64: 0.06%; 65 + : 0.73%		
	National and Regional (Chicago) A Model (2009 like) 0–4: 0.005%; 5–18: 0.01%; 19–29: 0.04%; 30–64: 0.06%; 65 + : 1.57%		
Cases averted (by age)	National and Regional (Chicago) B2 Model (1957 like) Varies by scenario. See Supplemental Table S1 for the National model and Tables S7 and S17 for the Regional Model	Effectiveness varied by scenario between best-case and worst-case scenarios from Germann et al., 2019	[5]^a,b^
	National and Regional (Chicago) B1 Model (1968 like) Varies by scenario. See Supplemental Table S3 for the National model and Tables S10 and S18 for the Regional Model		
	National and Regional (Chicago) A Model (2009 like) Varies by scenario. See Supplemental Table S5 for the National Model and Tables S13 and S19 for the Regional Model		
Diagnostic ratio/trigger for school dismissal	10%	5% and 20%	[5]^c^
Assumed vaccination rollout	Available 6 months after US index case, 2-dose schedule, 14 million doses per week	NA	[5]
Symptomatic fraction	67%	NA	[5]
Assumed vaccine efficacy (susceptibility)	70% for individuals < 65 years; 50% for individuals ≥ 65 years	NA	[5]
Assumed vaccine efficacy (infectiousness)	80%	NA	[5]
Number of students affected	Varies by scenario. Refer to Tables S4 (National Model), S7, S10, and S13 (Regional Models) for the fraction of schools closed	NA	[5]^a^
**Economic Model Parameters**			
Average daily wages	$190.88/day	NA	[9]
Average cost to treat (including those who do not seek treatment)^d^	National and Regional (Chicago) B2 Model (1957 like) 0–4: $271; 5–17: $127; 18–29: $373; 30–64: $476; 65 + : $9,293	± 25%	[8]
	National and Regional (Chicago) B1 Model (1968 like) 0–4: $205; 5–17: $117; 18–29: $180; 30–64: $262; 65 + : $2,053		
	National and Regional (Chicago) A Model (2009 like) 0–4: $306; 5–17: $170; 18–29: $263; 30–64: $348; 65 + : $2,272		
Average wages lost per case^d^	National and Regional (Chicago) B2 Model (1957 like) 0–4: $521; 5–18: $243; 19–29: $361; 30–64: $378; 65 + : $476	NA	[8]
	National and Regional (Chicago) B1 Model (1968 like) 0–4: $518; 5–17: $243; 18–29: $351; 30–64: $365; 65 + : $278		
	National and Regional (Chicago) A Model (2009 like) 0–4: $523; 5–17: $244; 18–29: $355; 30–64: $371; 65 + : $285		
Staff wages	$283.28/day (average mean wages for elementary and secondary schools divided by 180 days)	NA	[9]
Vaccination cost	Not calculated under the assumption that vaccination would occur with or without school closures	NA	NA
Staff-student ratio	1:8	NA	[10]
Average children per household	1.75	NA	[5]
Number of days out sick (children & workers)	1.6 days	1.6 – 5 days	[5]
Number of days out of school	2, 4, 8, and 12 weeks	NA	[5]
Fraction of households with children who missed work during previous unplanned school closures	20% (See Supplemental Information for details.)	5% – 40%	[11–16]
Fraction of schools able to switch to distance learning during closures	51.5% (See Supplemental Information for details.)	10% – 93%	[17–19]^e^
Nonwage benefits as a fraction of total wage + nonwage pay for employees	46%	NA	[20]
Discounted (3%) years of life-year lost per fatal case by age	0–4: 30.9; 5–18: 29.9; 19–29: 27.8; 30–64: 21.8; 65 + : 10.4	NA	[21]

^a^These parameters were summarized from a synthetic population developed in Germann et al., 2019. The model population was stochastically generated to match US and Chicago regional Census-based (Year 2000) distributions of age, household size, and employment status

^b^The best-case scenario was considered the baseline analysis in which children would experience a 50% decline in contacts outside their household and no change in contacts within their household. The worst-case scenario considered in the sensitivity analysis assumed that children would experience a 30% decrease in contacts outside the household and a 50% increase in contacts within their households

^c﻿^The diagnostic ratio/trigger for dismissal decision used in the model specifies the *fraction of symptomatic children with influenza infections* that would need to be identified to trigger school closures. With a lower diagnostic ratio/trigger, it takes longer for schools to begin to close because infected school children are detected more slowly (i.e., for a diagnostic ratio of 5%, only 5 out of 100 children with symptomatic influenza infections would be detected). Thus, schools would close once 20 symptomatic children were simulated in the model. In comparison, schools would be closed with ten symptomatic children for the 10% diagnostic ratio or five children with the 20% diagnostic ratio [5]

^d^This includes direct medical costs and out-of-pocket costs for individuals, who are not medically attended. These parameters were used for the National Model and the Chicago Regional B2 Model for the 1957-like pandemic, B1 Model for the 1968-like pandemic, and A Model for the 2009-like pandemic

^e^The lower bound estimate was based on data collected before the COVID-19 pandemic during which many schools were able to implement distance learning over time. By September 2020, 93% of respondents in households with school-age children had participated in distance learning [19]

Briefly, these epidemiological inputs are from an agent-based computational model, also known as Epidemiological Forecasting (EpiCast), designed to simulate community-level influenza transmission in the United States at the national scale [5]. EpiCast is a suite of stochastic, individual-based computer simulation models at the community (approximating a catchment area of a single school district, including elementary, middle, and high schools), regional (approximating a multi-county metropolitan area), and national levels. Each of these 3 geographic area models includes three key elements: a) community-level transmission through various social contact groups (including households, workplaces, schools, and others), b) disease natural history model and parameters, and c) Census demographics. The national model also includes a fourth element, namely: d) long-distance travel data from Bureau of Transportation [5]. A “baseline” model of pandemic spread in the absence of any mitigation measures can be adapted to incorporate pharmaceutical and non-pharmaceutical interventions to model how they affect person-to-person contact rates or the susceptibility, infectiousness, or disease course within individuals [5, 7]. The national-scale simulation model consists of 281 million individuals distributed among 65,334 census tracts to closely represent the actual population distribution according to publicly available 2000 US Census data [5]. Each tract is in turn organized in 2,000-person communities resulting in 180,492 model communities. The model combines US Census demographics and worker-flow data to generate daytime and evening contact networks based on potential contacts emerging at schools, workplaces, households, neighborhoods, and communities [5]. Transmission within each contact group is described by a contact probability, which may depend on the age of both the infectious and susceptible persons. Individual contact probabilities were adjusted to be consistent with age-stratified attack rates and infection sources [5, 7]. When schools are closed, the model assumes a 50% reduction in the number of child-related contacts outside of the household and no change in the number of household contacts. The lower bound estimate of effectiveness instead assumed a 30% reduction in child-related contacts outside the household and that child-related household contacts would double.

The national model uses 3 geographic scales for PSC decisions including “community,” “county,” and “multi-county,” which correspond to closing schools one at a time (i.e., community), for the entire school district (i.e., county), or for a group of adjoining counties (i.e., multi-county) once the dismissal decision is reached for any given school. This means that the multi-county closure area is the most aggressive decision to prevent the spread of the pandemic in terms of the number of schools closed. Closure duration scenarios included 2 weeks, 4 weeks, 8 weeks, and 12 weeks. Our study considered the cost of each of these intervention strategies as simulated by the EpiCast model [5]. The model also examined different triggers for school dismissal based on the fraction of symptomatic children that would cause closures to occur. The trigger is linked to assumptions about the sensitivity of the surveillance system used to detect infected schoolchildren based on the diagnostic ratio. The diagnostic ratio is based on the fraction of symptomatic children infected with influenza who would be detected. With a lower diagnostic ratio/trigger, it takes longer for schools to begin to close because infected school children are detected more slowly (i.e., for a diagnostic ratio of 5%, only 5 out of 100 children with symptomatic influenza virus infections would be detected by the surveillance system). This paper focused on an assumed diagnostic ratio of 10% and provided estimates for 5% and 20% diagnostic ratios in the sensitivity analyses and supplemental information. The geographic area corresponds to the population from which an infected schoolchild would be detected (i.e., one child within the community for community-level closures versus one child in the multi-county area for the multi-county-level closure. If school closures occur as soon as a single child is diagnosed with pandemic influenza, diagnostic ratio/triggers of 5%, 10%, and 20% would require 20, 10, and 5 symptomatic children respectively within each geographic area. More information on daily contact probabilities and sources of infection used to calibrate the model can be found in the supplemental information accompanying the articles describing the transmission models [5, 7].

### Net economic cost of preemptive school closures

We compute the (net) economic cost of the intervention based on the total cost of PSCs minus the averted cost of pandemic cases due to PSCs. The cost of PSCs was derived from the wage losses of school staff due to the schools closing and the wage losses of parents who must care for children out of school. The wage losses of school staff from schools closing include costs for school employees, who are assumed not to work during the closure when the schools do not provide synchronous distance learning during closures. The economic costs include lost productivity even if the school employees are paid during the closure. We developed a proxy to estimate this lost productivity based on the wages of school employees multiplied by the duration of school closure. We applied a correction factor (base-case: 51.5%, range: 10% to 93%) called *fraction of distance learning* to account for schools that can provide education through online learning to offset some of the lost productivity caused by the school closure based on the pre-COVID-19 baseline (see supplemental information). During the COVID-19 pandemic, about 93% of households with school-age children had participated in distance learning by September 2020 [19]. This represents an upper bound estimate of the number of students who may be able to access distance learning in the event of a future influenza pandemic. However, this may be an over-estimate for schools to implement closures for shorter-term closures of between two and twelve weeks for pandemic influenza. A lower bound estimate (10%) is based on survey data regarding the fraction of schools with distance learning capacity prior to the COVID-19 pandemic. The future potential for distance learning to be implemented will likely fall between these bounds as some schools will maintain capacity for distance learning, but many schools may be unable to transition immediately from in-person learning to distance learning. In addition, some studies have shown a decline in student achievement as a result of the COVID-19 pandemic, which may be associated with the replacement of in-person schooling with distance learning [17]. To account for this uncertainty, we used the midpoint of the two previously reported fractions as the most likely estimate and considered a range between the lower and upper bounds for the uncertainty analysis.

To estimate the cost of parents’ lost wages, we first divided the number of affected children by the average number of children per household (1.75) to estimate the number of affected households. The number of households with children was then multiplied by the *fraction of households with children whose parents missed work* to estimate the number of parents missing work. Next, the estimated number of parents missing work was multiplied by the US average hourly wage rate with non-wage benefits, the duration of school closures, and an assumption of 8 h worked per day.

Not all parents would need to miss work to care for children during PSCs. To estimate the *fraction of households with children whose parents missed work,* we reviewed published studies that reported the fraction of parents that missed work due to unplanned school closures in response to the 2009 H1N1 pandemic or due to local excess absenteeism from elevated numbers of influenza-like-illness in US communities (see supplemental information). These studies found that adults from between 14 and 29% of households had missed time at work due to school closures [11–16]. One of the studies was a national survey of households impacted by school closures during the 2009 H1N1 pandemic and found that adults in 20% of households had at least one parent who missed work (Table S2). Among households in which an adult had to miss work, we assumed that one parent or guardian from each family would be at home to care for the child(ren). The base-case scenario assumed that 20% of households (range 5% to 40%) would have at least one parent who would miss time at work to care for children during PSCs. We used a conservative estimate by assuming that one adult in each of these households would miss work during the full period of the school PSC. The sensitivity analysis as well as the regional “deep dive” explores variations of this parameter (see supplemental information). This factor helps to account for families that include older children or other non-working adults (e.g., retired grandparents) that would be able to supervise younger children as well as non-working parents or parents with access to telework, staggered work schedules, or other means to reduce the economic costs to households from lost time at work.

The effectiveness of each intervention was calculated based on the percentage reduction in the expected number of cases relative to no intervention as reported previously [5]. The averted cost of pandemic cases from PSCs includes treatment costs of cases averted, productivity loss of ill workers associated with averted cases, and productivity loss of parents of ill children. The treatment costs were defined to include direct medical costs incurred from visiting healthcare providers and estimated out-of-pocket costs for non-medically attended cases. The age-specific treatment costs of cases averted were estimated based on a recent analysis of the costs of pandemic influenza in the United States using insurance claims data [8]. The productivity loss of ill workers was estimated for the numbers of cases averted among the working adult and senior populations. The cases averted for the working adult and senior populations were multiplied by average daily wages (the US average hourly wage rate with an assumption of 8 h worked per day considering non-wage benefits) and number of days out because of illness. The productivity loss of parents of ill children was estimated by multiplying numbers of cases averted due to PSC for children 12 years old or younger, fraction of working adult population, average daily wages including non-wage benefits, and number of days out due to taking care of ill children. All costs are presented in 2016 US dollars (USD). More details are provided in the supplemental information.

We estimated the numbers of deaths averted based on the numbers of cases averted as a benefit of the intervention. The number of deaths averted was calculated by multiplying the number of cases averted by the age-specific case fatality rate [8]. Using these age-specific case fatality rates results in a higher overall case fatality rate than the 0.1% rate used in Germann et al. [5]. This assumption was made because the present age distribution in the United States is considerably older than in 1957, when this pandemic occurred, and the case fatality rate in the > 65 years age group was much higher than for other age groups (3.81%). The number of life-years gained is calculated by multiplying the number of deaths averted by age group by the average U.S. life expectancy for each age group [21] after discounting future life-years using a 3% annual rate. We calculated the number of discounted life-years saved to present results that account for the much higher case fatality rate in this older population for the 1957-like pandemic scenario. This metric may also be used for comparison with other pandemic scenarios for the regional model. Specifically, the 1968-like scenario had much lower case fatality rate estimates for all age groups and the 2009-like scenario was not as skewed to the elderly in terms of case fatality rates (Table 1).

The incremental cost-effectiveness ratio (ICER) of each intervention was assessed by calculating the net cost per case/death averted and the net cost per life-year gained. The net economic cost of the intervention was divided by the change in health outcomes to calculate each ICER. The ICERs were calculated relative to no intervention and incrementally based on the duration of closure. The relative efficiency of interventions is evaluated based on whether the ICER is larger or smaller for one intervention strategy compared to another such that the intervention.

We also analyzed the impact of PSCs for three hypothetical influenza pandemics with viruses with transmissibility and clinical severity similar to those of the 1957 (referred to as 1957-like), 1968 (1968-like), and 2009 (2009-like) influenza pandemics using a regional model of the Chicago region as previously reported [5]. The input parameters for the regional models are generally like those of the national-level model, except for accounting for different attack rates and severity for each pandemic influenza virus and using a regional rather than national model of the population impacted. Key parameters are summarized in Table 1. The regional analysis only considered 2 geographic scales: individual schools (i.e., community) and regional (i.e., multi-county). The regional model is like the multi-county approach used in the national analysis. However, since the Chicago region itself is one multi-county region, in practice, the regional approach would lead to the simultaneous closure of all schools once triggered.

### Sensitivity analysis

We conducted multiple univariate sensitivity analyses to account for uncertainties in the cost of PSCs, the value of averted cases, the effectiveness of PSCs in reducing the number of cases, and the case fatality rate. The estimated effect of each parameter on the net cost of the intervention was plotted to demonstrate the relative importance of uncertainty resulting from each parameter. The effectiveness of PSCs varied from a “worst-case” impact on transmission with a doubling of assumed contacts among individuals within a household and only a 30% reduction in contacts among children across households relative to the “best-case” scenario. In the best-case scenario, there is no increase in household contacts and a 50% reduction in contacts among children from different households. The “best-case” scenario is shown as the “base-case” scenario in this analysis.

## Results

**Fig. 1:**
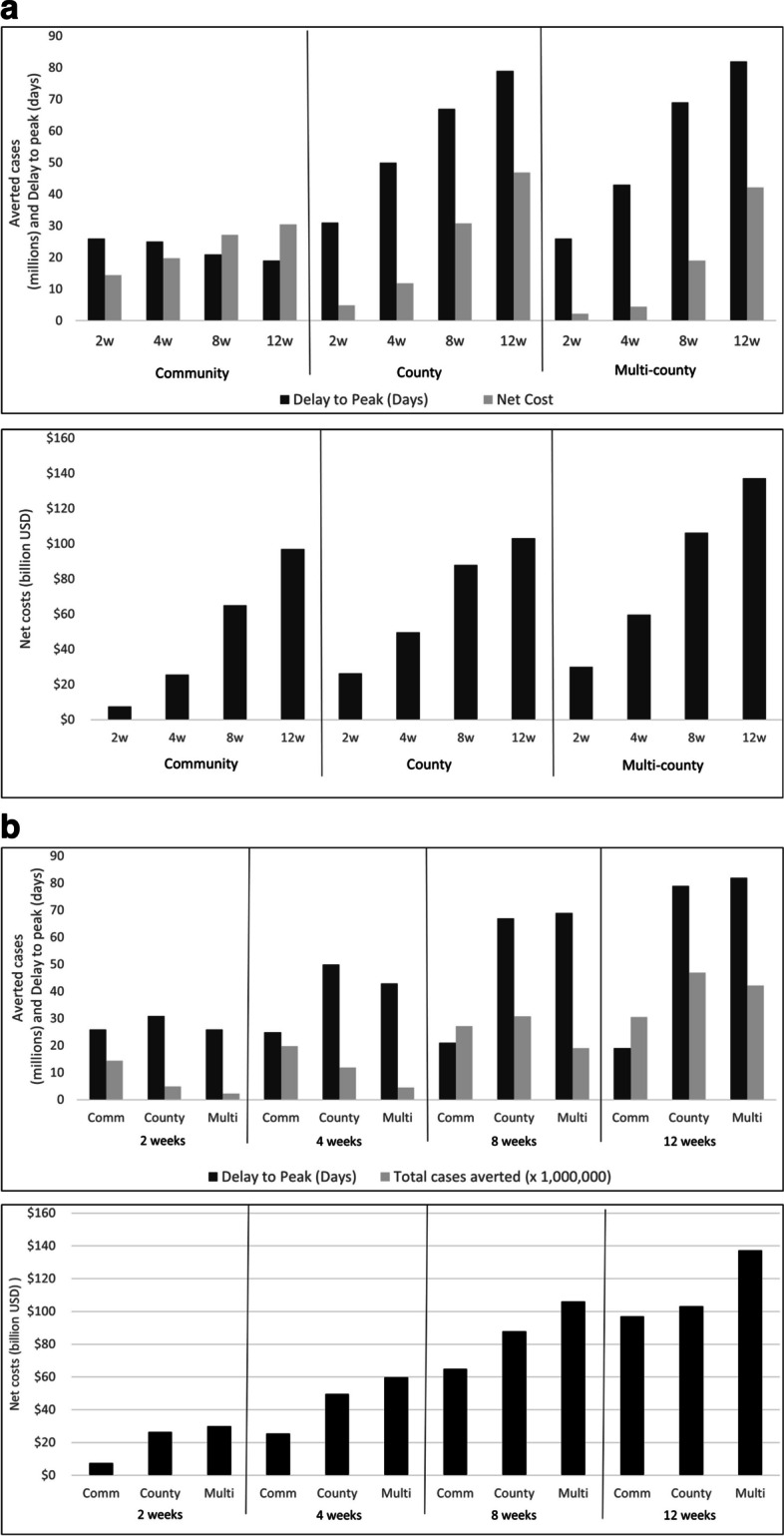
**a** National-level effect of increasing school closure duration on cases averted, delay to peak, net cost*. **b** National-level effect of increasing geographic scale of school closures on cases averted, delay to peak, net cost*. *Within each subdivision, geographic scale (1**a**) or duration (1**b**) of school closures is held constant for a national-level strategy against an influenza pandemic similar to the 1957 pandemic. This figure is based on the 10% diagnostic ratio for school closures under the assumption that only 10% of symptomatic school children infected with influenza would be detected by a symptom-based surveillance system and schools would close when the first symptomatic child was diagnosed [5]

Focusing first on the PSCs that were triggered under the 10% diagnostic ratio assumption for the national model (1957-like), the net costs of the PSC increased from $7.5 billion for 2-week community closures to $137 billion for 12-week multi-county closures (Fig. 1a). The net costs increased significantly with the duration of the closure and for larger geographic scales of closures. The benefits in terms of the number of cases and deaths averted through PSCs varied from 2.3 million cases and 7,100 deaths averted for the 2-week multi-county closures to 47 million cases and 156,000 deaths averted for the 12-week county closures (Table S3). The duration of closure had a larger impact on the number of cases averted for both the county and multi-county closures areas than for the community closures. For example, in doubling the closure duration from 4 to 8 weeks, the number of cases averted increased by 2.6 and 4.2 times for the county and multi-county closures, but only by 1.6 times for the community closures.

Figure 1b shows that the net cost was consistently lower for the community closures for an equivalent duration and that the number of cases averted was highest for the community closures for durations less than or equal to 4 weeks. However, the number of cases averted was greatest for county closures for durations of eight weeks or greater. The multi-county closures consistently had the highest costs and fewest cases averted for a given duration except for the 12-week duration for which the number of cases averted was greater than for the community closures. For durations greater than 2 weeks, the community closures had less impact on the delay to peak incidence relative to county or multi-county closures. The longest delay to peak (82 days) resulted from a 12-week, multi-county closure strategy. This compares to 79 days for a 12-week county closure strategy or 19 days for a 12-week community closure strategy.

**Fig. 2:**
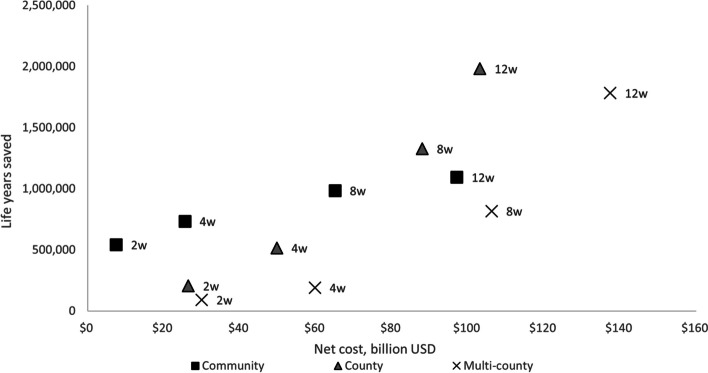
Net cost (billion USD) and number of life-years gained for community, county, and multi-county school closures*. Notes: The duration of school closures increases from 2 weeks to 4 weeks to 8 weeks to 12 weeks for community closures (square), county closures (triangle), and multi-county closures (x). The net costs and life-years gained increase with the durations of closures for each geographic scale. This figure is based on the 10% diagnostic ratio for school closures under the assumption that only 10% of symptomatic school children infected with influenza would be detected by a symptom-based surveillance system and schools would close when the first symptomatic child was diagnosed [5]

The net costs and numbers of life-years gained for each intervention for the national-level model are shown in Fig. 2 and the net costs per discounted life-year gained for each intervention are summarized in Supplemental Information Tables S4 and S5. The most efficient intervention was the 2-week community closure, with a net cost per discounted life-year gained of about $14,000 relative to no intervention. This would be the best alternative to support an initial assessment of the transmissibility and clinical severity of the pandemic virus (Objective 1 above). For community closures, the net cost per discounted life-year gained increased with duration from $14,000 for 2 weeks to $89,000 for 12-week closures (Supplemental Information Table S4). The incremental cost per discounted life year gained from increasing the duration of community closures increased from $94,000 (4 weeks vs. 2 weeks) to $295,000 (12 weeks vs. 8 weeks) (Supplemental Information Table S5). This demonstrates decreasing returns to scale for increasing the duration of community closures. In contrast, the net cost per discounted life-year gained decreased from 2-week closures ($129,000 for county closures and $331,000 for multi-county closures) to 12-week closures ($52,000 for county and $77,000 for multi-county) such that 12-week durations for both county and multi-county closures would be more efficient than community closures (Supplemental Information Tables S4 and S5). The increasing returns to scale suggest that county closures became more efficient in terms of the net cost per discounted life-year gained with increasing duration from 2 to 12 weeks. The numbers of cases averted and life years gained were maximized with 12-week county closures (Objective 3 above).

**Fig. 3:**
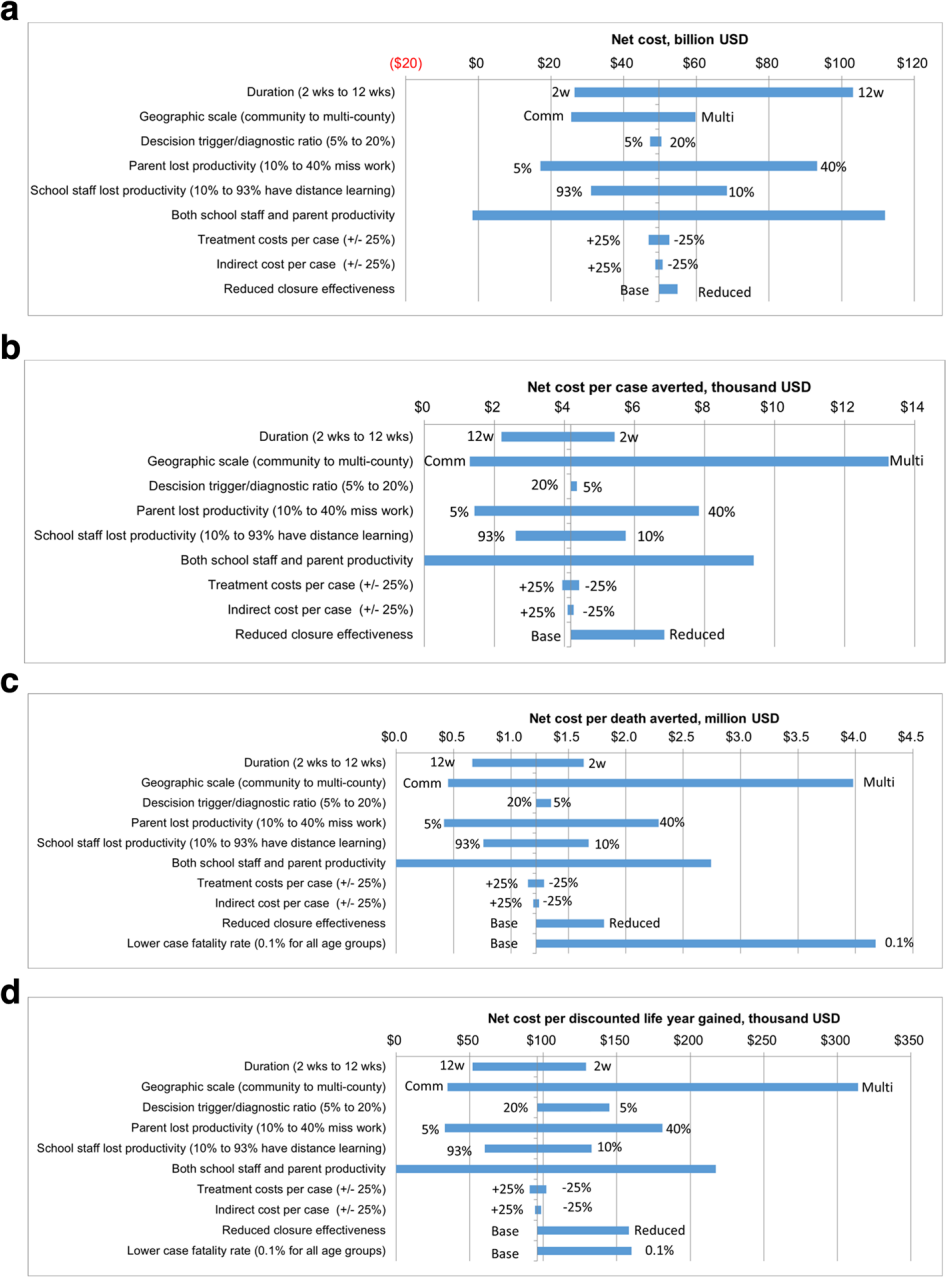
**a** One-way sensitivity analyses of school closure net cost, base 4-week county closure, 10% diagnosis ratio*. **b** One-way sensitivity analyses of net cost per case averted, base 4-week county closure, 10% diagnostic ratio*. **c** One-way sensitivity analyses of net cost per death averted, base 4-week county closure, 10% diagnosis ratio*. **d** One-way sensitivity analyses of net cost per life-year gained, base 4-week county closure, 10% diagnosis ratio*. * Baseline estimates: Duration 4 weeks; Geographic scale County; Parent lost productivity 20%; School staff lost productivity: 51.5% (i.e., 51.5% have access to distance learning); Base case estimates: $49.7 billion net cost, $4,180 per case averted, $1.22 million per death averted, $96,000 per life-year gained. This figure is based on the 10% diagnostic ratio for school closures under the assumption that only 10% of symptomatic school children infected with influenza would be detected by a symptom-based surveillance system and schools would close when the first symptomatic child was diagnosed [5]. Treatment costs were defined to include direct medical costs incurred from visiting healthcare providers and estimated out-of-pocket costs for non-medically attended cases. Figures 3b-3d are included with the x-axis truncated at zero because there is a scenario in which net cost savings are achieved (see Fig. 3a). When net cost savings are achieved, the intervention would be considered dominant relative to no intervention

A series of one-way sensitivity analyses of the net cost and net cost per case averted, per death averted, and per life-year gained are shown in Fig. 3a through 3d. Relative to county closures with a 4-week duration, shorter durations were less costly but also less efficient, and longer durations were more costly and more efficient. At 4 weeks, a shift from county to community closures would increase efficiency, while a shift to multi-county–level PSCs would reduce efficiency (i.e., increase the net cost per case/death averted or life-year gained). The decision trigger/diagnostic ratio had less effect on effectiveness or efficiency relative to duration or geographic scale. The assumed diagnostic ratio had the greatest impact on community closures (Supplemental Information Table S3 and Figures S1a-c and S2a-c) such that the 20% trigger was much more effective and the 5% trigger much less effective than the 10% trigger.

Lost productivity for parents during PSCs resulted in more uncertainty than the fraction of schools with distance learning capabilities, which affected lost productivity of school staff. The net cost for a 4-week, county closure varied from $17 billion to $93 billion (base-case: $68 billion) depending on the fraction of parents that would miss time at work due to the closures. In contrast, uncertainty in the fraction of schools with distance learning capacity only resulted in a range of $31 to $68 billion. In comparison to the costs of closure, the uncertainty in averted costs (benefits) from averted illnesses contributed to less uncertainty in net cost and efficiency estimates.

If PSCs were less effective than assumed in the base-case, the net cost per case averted would increase from $4,200 to $6,900, the net cost per death averted would increase from $1.2 million to $1.8 million, and net cost per discounted life-year gained would increase from $96,000 to $160,000 for a 4-week, county closure. Another source of uncertainty in the net cost per death averted and per discounted life-year gained was the case fatality rate such that the net cost per death averted would increase to $4.2 million and the net cost per discounted life-year gained would increase to $160,000 if a constant 0.1% case fatality rate were applied to all age groups.

**Fig. 4:**
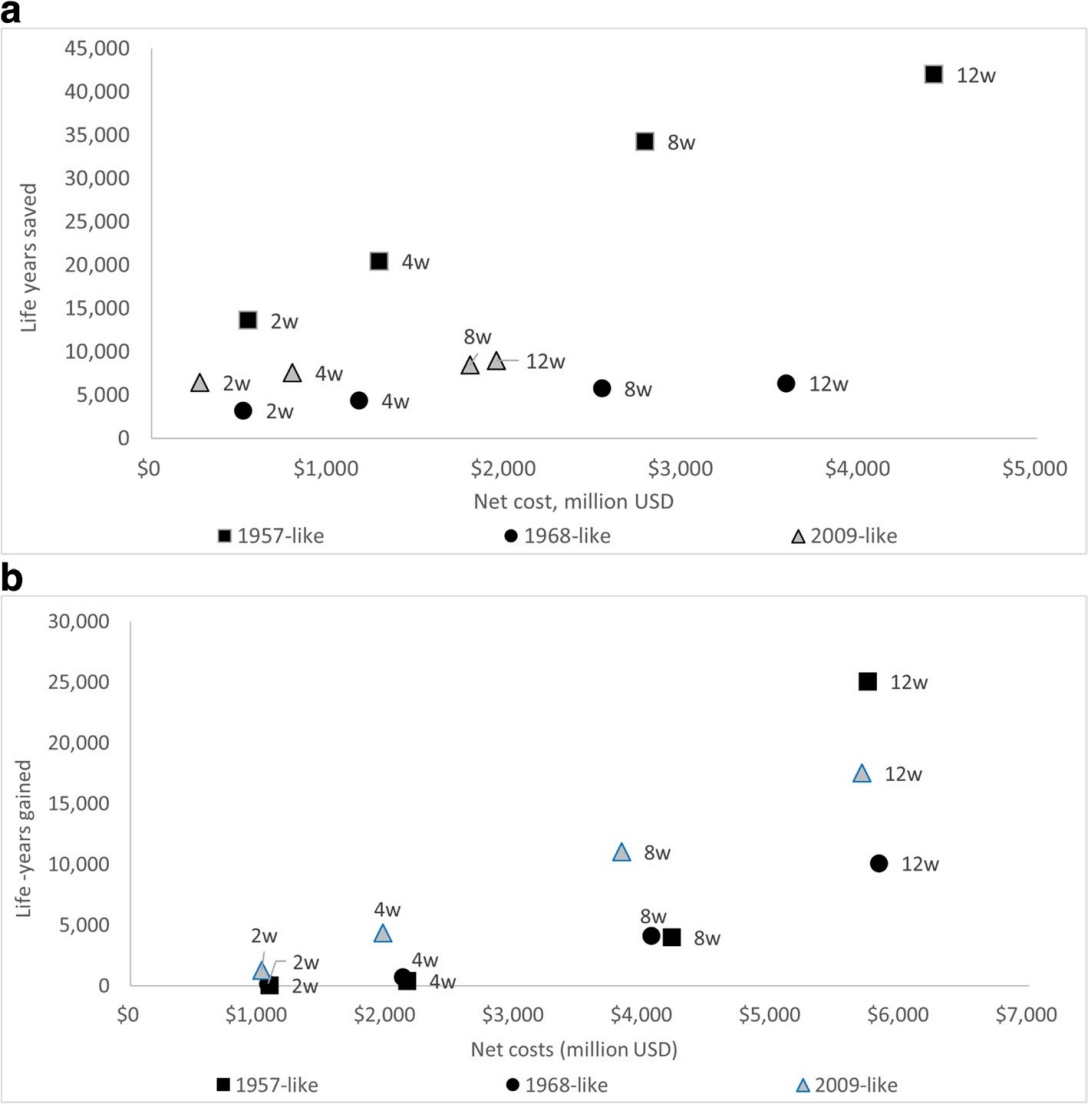
**a** Net cost (billion USD) and life-years gained for community school closures during for three hypothetical influenza pandemics*. **b** Net cost (billion USD) and life-years gained for regional school closures for three hypothetical influenza pandemics*. * This figure is based on the 10% diagnostic ratio for school closures under the assumption that only 10% of symptomatic school children infected with influenza would be detected by the surveillance system and schools would close when the first symptomatic child is diagnosed [5]. Treatment costs were defined to include direct medical costs incurred from visiting healthcare providers and estimated out-of-pocket costs for non-medically attended cases

For the Chicago regional analysis, we examined three different pandemic scenarios (but only two geographic scales). The impact of PSC appears generally less cost effective for the regional model since the cases are always seeded within the region at the outset of the analysis period and thereafter quickly spread throughout the region. In contrast, in the national model cases were seeded in various places and some simulated pandemics may not spread throughout the country within the 180-day period prior to the assumed initiation of vaccination campaigns. The estimated net cost per discounted life-year gained were considerably higher for the regional model using the 1957-like pandemic than for the national model (e.g., Chicago region: net cost of $40,000 to $105,000 per discounted life-year gained for community closures with a 10% dismissal trigger versus $14,000—$89,000 for the national model). The net cost per discounted life-year gained from multi-county (regional) closures with a 10% dismissal trigger in the Chicago area exceeded $1 million for all durations less than 12 weeks. The estimated number of life-years gained from regional closures increased non-linearly such that an 8-week closure duration results in almost 10 times more life-years gained than a 4-week closure and a 12-week closure results in more than six times more life-years gained than an 8-week closure (Fig. 4a-b). Thus, the regional closure strategy was relatively ineffective for durations of 8 weeks or less and the marginal cost per life-year gained decreased as the duration of closure increased. The number of life-years gained was higher and the net cost was lower for the 2009-like pandemic than for the 1968-like pandemic across all the regional analyses. The cost per life-year gained was therefore higher for the 1968-like than the 2009-like pandemic.

Community closures were much more effective than the regional closures for any duration using the regional model. Considering community closures for the 1968-like and 2009-like pandemics, the number of life-years gained and savings from averted cases from PSCs were lower than for the 1957-like pandemic because of lower transmissibility and severity for these pandemics. The marginal improvement in the number of life-years gained was limited for durations greater than 4 weeks for 1968-like and 2009-like pandemics. For example, the marginal net cost per life year gained increased from $160,000 for 2 -week closures vs. no closure to $1.7 million for 12-week vs. 8-week closures for the 1968-like pandemic (Supplement Table S11). For the 2009-like pandemic, the marginal net cost per life year gained increased from $42,000 for 2-week versus no closures to $1.1 million for 8-week vs. 4-week closures (Supplement Table S14). The community closures resulted in lower net costs than the regional closures for all three pandemics and the net cost per discounted life-year gained for community closures with a 10% dismissal trigger exceeded $40,000 for all interventions. Refer to Supplemental Information, Tables S6-S14 and Figures S1a-c and S3a-b, S5, and S6.

## Discussion

There are important considerations to the decisions made in closing schools preemptively in response to an influenza pandemic. With larger geographic scales and longer durations of PSCs, their costs dramatically increase relative to shorter duration community closures. The net costs of closures with 10% dismissal trigger vary from $7.5 billion for 2-week community (school-by-school) closures to $137 billion for 12-week multi-county closures for the national-level model. However, as the costs of closures increase, the benefits in terms of cases and deaths averted also increase from 2.3 million cases and 7,100 deaths averted for 2-week multi-county closures to 47 million cases and 156,000 deaths averted for 12-week county closures. This congruence of the directions between increasing costs and in parallel increasing benefits of preemptive school closures underscores the importance of optimizing the duration and geographic scale of preemptive closures for the desired public health objective. The net cost per death averted varied between $190,000 and $4.2 million for the national-level model. In comparison, the U.S. Department of Health and Human Services (HHS) has recommended the use of values of between $4.6 and $15.0 million (2016 USD) of the value of statistical life for regulatory impact analyses [22]. The net costs per death averted for all strategies were less than the HHS-recommended lower-bound value of statistical life, indicating that mortality reduction benefits of PSCs would exceed the estimated costs of closures for a pandemic like that which occurred in 1957.

In our model, county closures approximate school district closures, because most US counties have a single public K-12 school district. Compared to community (i.e., single school) closures, the county closures are equally or more cost-efficient (in terms of net cost per death averted) for durations ≥ 8 weeks and maximize the number of cases and deaths averted (Fig. 2). Additionally, in the United States, school district-wide closures are much more frequent than closures of individual schools [23, 24], likely reflecting the programmatic considerations as well as the organizational level that usually authorizes such a drastic measure. In general, both county and multi-county closures are associated with greater delays to peak incidence (i.e., flattening the curve), albeit at higher net costs than community closures. County closures have lower net costs as well as more cases/deaths averted compared to multi-county closures with similar though slightly shorter delays to peak incidence.

Community closures were estimated to be considerably more cost-efficient in terms of the net cost per case or death averted for shorter durations (≤ 4 weeks). This geographic scale of closure in our model is most congruent to school-by-school decision making, where each individual school (rather than an entire school district) would decide on its own to close. In a potential pandemic, this lowest level of PSCs may be both pertinent and appropriate very early in an evolving pandemic; for example, if a school-associated case or cluster of cases of unsubtypable influenza A is recognized quickly, this will prompt a local investigation to determine the epidemiologic features of the outbreak. However, in practice, such a scenario is highly unlikely, since it would require each individual school to have the capability to promptly and accurately detect and diagnose cases of novel (i.e., unsubtypable) influenza A among students and staff. Sensitivity analyses suggest that the number of cases averted would increase with more sensitive surveillance, reflected in a higher trigger (20%) for dismissal decisions for community closures. The net cost per life-year gained would decrease from a range of $14,000 to $89,000 for community closures with a 10% trigger to $3,500 to $25,000 with a 20% trigger. However, the dismissal decision is less important for county or multi-county closures. This suggests that the sensitive and timely surveillance needed for prompt triggering of the intervention is especially important for the effectiveness and efficiency of the community PSC approach, but less important for the county and multi-county approaches.

The net cost per case averted varied from about $500 to $6,000 for most scenarios except for the multi-county–level closures for ≤ 4 weeks for which the net cost per case averted was greater than $13,000. The net cost per death averted varied from $0.18 to $1.2 million for community closures, $0.66 to $1.6 million for county closures, and $0.98 to $4.2 million for multi-county closures. The net cost per life-year gained varied from $14,000 to $89,000 for community closures, $52,000 to $129,000 for county closures, and $77,000 to $331,000 for multi-county closures. These estimates can be compared to regulatory guidance for the valuation of a quality adjusted life-year gained, which vary from $230,000 to $750,000 [22].

Not surprisingly, the net cost per life-year gained was much higher for the less severe pandemics (1968-like and 2009-like scenarios) compared to the 1957-like pandemic, as summarized in the Supplemental Information. At the regional level, the regional closure strategy is only moderately effective for durations less than 12 weeks for the 1957-like and 1968-like pandemics. The number of life years saved was consistently greater for the 2009-like pandemic than for the 1968-like pandemic. At the regional level, it appears that the more targeted community closures are more effective, especially at shorter durations. This suggested that highly targeted closures could be especially effective in the regional context if surveillance capabilities were sufficient to be implemented. Given the significant costs associated with PSCs, while influenza pandemic severity is still unknown, it is prudent to close schools for shorter periods (e.g., 2 weeks) to help gather the data needed to ascertain more information about the specific virus. To the extent possible, during influenza pandemics PSCs should be executed at the smallest possible geographic scales (e.g., individual schools or school districts). At the wider geographic scale (regional / multi-county), short-term closures (e.g., 2 weeks) were estimated to have limited cost effectiveness for reducing influenza burden; the net cost per discounted life-year gained with a 10% dismissal trigger was estimated to be $5.8 million for the 1968-like pandemic and $0.8 million for the 2009-like pandemic. In contrast, the estimated net cost per life-year gained for 2-week, community (school-by-school) and school district closures were $159,000 for the 1968-like and $42,000 for 2009-like pandemics.

A limited number of studies have attempted to quantify the potential economic costs associated with school closures for a specific region and/or pandemic severity. For example, Sadique et al. [25] estimated the potential economic cost associated with school closures during mild to severe pandemics (i.e., ranging from 2 to 12 weeks) in the United Kingdom. Their findings show that closures could result in 16% workforce absenteeism and £0.2 to £1.2 billion costs per week. Lempel et al. [26] estimated the potential economic costs associated with closing all schools in the United States for 2 to 12 weeks as a response to mild to severe pandemics. Their findings show that closing all schools for a 4-week period could result in $10 to $47 billion dollars in costs among households with school-age children and a reduction of 6% to 19% in key health personnel. The estimated costs to households in this analysis fell within the range estimated by Lempel et al., although overall costs were higher in our analysis because we included costs to school staff. Brown et al. [27] used an agent-based simulation to explore school closures in Pennsylvania ranging between 1 and 8 weeks during the 2009 H1N1 pandemic. Their findings showed that closing schools for 8 weeks would have resulted in median net costs of $21 billion (95% range: $8 to $45.3 billion). They concluded that the cost associated with school closures might have outweighed the cost savings in preventing influenza cases during the 2009 pandemic. These cost estimates included lost wages for households and lost productivity for school employees and were significantly higher than our cost estimates.

Our findings should be considered in context of several potential limitations. First, the costs of PSCs on the schools and school employees as well as the parents of school children were difficult to estimate because of their unprecedented nature. Specifically, large-scale PSCs had not been attempted in the United States to control infectious disease outbreaks until the COVID-19 pandemic in 2020. We estimated these parameters based on observations from short-term school closures during previous influenza outbreaks and attempted to account for uncertainty by conducting a sensitivity analysis of costs for schools and the parents of school children. One strength of this analysis is the incorporation of empirical data on the impacts of PSCs on parents’ abilities to continue working even if the data are limited to short-term closures. About 93% of people in households with school-age children had participated in distance learning by September, 2020 [19]. However, it is difficult to predict whether school districts will maintain this capacity in the future. Maintenance of distance learning capacity and improvement in telework capabilities for parents would reduce the costs of PSCs as demonstrated by the lower bound values shown in Fig. 3a-d.

Second, treatment costs are based on data from more recent influenza outbreaks and estimated differences in hospitalization rates between the 1957 pandemic and more recent outbreaks, which were much less severe than the 1957 pandemic [8]. The potential case fatality rate for future influenza pandemics also remain uncertain. On the one hand, the effectiveness of treatment for influenza has improved since 1957. Yet, the age distribution for the US population has shifted upward such that a greater fraction of the total population is in the older, higher-risk category for severe influenza illness. In the sensitivity analysis, we analyzed a flat 0.1% case fatality rate across age groups as well as the population-weighted case fatality rates presented in Table 1. However, even with such a relatively low assumed pandemic case-fatality ratio, our results are consistent with other published model-based analyses which have examined hypothetical outbreaks with higher case fatality rates [28, 29]. The number of life years lost was estimated based on the average life expectancy by age group; however, the risk of death from influenza increases for patients with medical comorbidities [30], who would also be expected to have shorter life expectancies relative to the average for each age group.

The third limitation is related with the fact that the ability to forecast the spread of a transmissible disease and its severity is limited to data from past pandemics and hence may not be accurate. This study relies on historic information and data associated with only one virus of pandemic influenza, the 1957 influenza A (H2N2), for the national model, and three pandemic influenza viruses (1957-like, 1968-like, and 2009-like) for the regional model, and a limited sensitivity analysis around the effectiveness of school closures. One strength of the present analysis is the use of an agent-based model at the national scale, which incorporates demographic and spatial heterogeneities at sub-regional levels and enables head-to-head comparisons between multiple geographic scales of closures. However, as noted in the paper in which this transmission model was first reported [5], all mathematical models of disease transmission are limited by their necessary assumptions and the availability of data to parameterize the model. Although the authors attempted to consider and address many of these limitations, they identified a few key issues. First, it is difficult to model how contact rates (within different mixing groups and ages) would change after an unplanned school closure. Over time, at least in some settings such changes may revert back to mixing patterns similar to those pre-closure rates depending on each community’s perceived severity of illness. Presumably, this may be especially true for scenarios where pandemic influenza may be associated with real or perceived lesser severity (e.g., the 1968-like and 2009-like scenarios considered in the regional models had an objectively (i.e., real) lesser severity than the 1957-like scenario). In addition, there remains considerable uncertainty in the natural history of influenza; our analysis projects the outcomes of past pandemics that may or may not correspond to future influenza pandemics [5]. The authors also noted that the findings from this model were consistent with previous studies considering school closures during influenza pandemics, and that they believed their analysis was the most comprehensive modeling study to consider effectiveness of different school closure strategies to mitigate influenza in the United States during an ongoing influenza pandemic [5, 7]. PSCs reduce transmission among school-age children, who could otherwise infect members of their households and other age groups within their communities. Thus, even if older grandparents were to supervise children during PSCs, these grandparents would be less likely to have contact with an infected grandchild even if they increased their total number or duration of contacts with a school-age grandchild during PSCs.

Fourth, PSCs also may result in unintended consequences not captured in this study due to the limited data available to parametrize the model. For example, some parents have expressed concerns about arranging childcare during prolonged school closures, which could result in job losses. Academic performance (e.g., standardized test scores) in districts affected by prolonged school closures may be lower, potentially affecting future student placement [31]. For students enrolled in school meal programs, school closures may cause additional financial burden for their families as some affected students may miss meals that they would get at school, even as the vast majority of parents interviewed (> 96%) support the decision to close schools [11]. Loss of other school-based services, such as counseling or specialized support for some students with disabilities, may disproportionally impact certain vulnerable groups of students who rely on them. School closures also introduce equity concerns regarding the abilities of different socio-economic groups to cope with closures [32, 33] or experience disproportionate learning loss [17], which deserves attention as millions of students in the United States and worldwide had to study online, for prolonged periods of time in 2020–2021, during the COVID-19 pandemic caused by a novel coronavirus, SARS-CoV2. These and any other presumed unintended consequences and potential coping mechanisms that limit their detrimental impact on children and families are difficult to quantify due to limited data to inform these potential outcomes. As more data are generated on job losses associated with the need to provide childcare during PSCs and learning loss associated with closures, it would be worthwhile to revisit this analysis to better account for these costs. The widespread adoption of distance learning during the COVID-19 pandemic may ameliorate some of the costs associated with learning loss if PSCs are implemented in the future as we considered in the sensitivity analysis. The impact of potential job losses is more difficult to quantify because it would require an assessment of how long parents or other caretakers would have to leave the workforce. Further, the COVID-19 pandemic caused significant disruptions to the job market in the United States, which complicates a marginal analysis of the impact of PSCs on the parental job losses. One analysis of found that although mothers of young children, especially those without a four-year college degree were more likely to lose jobs during the COVID-19 pandemic, the overall effect on parents of young children (less than 13 years) was not statistically significant after accounting for characteristics including age and education [34]. We additionally note that the duration of closures and distance learning considered in this analysis are of shorter duration than many districts’ closures or distance learning efforts during the COVID-19 pandemic.

## Conclusion

We found that closing schools by county (or school district) for longer durations (8 to 12 weeks) would result in the most cases (31–47 million) and deaths (105,000—156,000) averted, albeit at considerable cost ($88-$103 billion net of averted illness costs) for a national-level model of a 1957-like pandemic. The net cost per death averted was estimated to be between $660,000 and $841,000 and the net cost per life-year gained between $52,000 and $66,000 for these scenarios. These estimates compare very favorably to the range of value of statistical life estimates recommended for regulatory impact analyses ($4.6 to 15.0 million) suggesting that the benefits of preemptive school closures to mitigate influenza pandemics of varying severity would exceed the costs. We also found closing schools individually for 2-week periods had the lowest cost per discounted life-year gained ($14,000). The finding supported that community closures are an attractive alternative at the outset of an outbreak, while attempting to assess the transmissibility and severity of a new pandemic influenza virus. These estimates can assist decision makers assess the cost and benefits of PSCs in response to influenza pandemics.

## Disclaimer

This work was sponsored by the Centers for Disease Control and Prevention. Los Alamos National Laboratory, an affirmative action/equal opportunity employer, is operated by Triad National Security, LLC, for the National Nuclear Security Administration of the U.S. Department of Energy under contract # 19FED1916814CKC. Because this research did not involve human subjects, it was not subject to IRB review requirements.

The findings and conclusions in this report are those of the authors and do not necessarily represent the official position of the Centers for Disease Control and Prevention.

### Supplementary Information

**Additional file 1.**

## Data Availability

All data generated or analyzed during this study are included in this published article and its supplementary information files.

## Abbreviations

NPIs: Nonpharmaceutical interventions
PSCs: Preemptive school closures
CPSTF: U.S. Community Preventive Services Task Force
NA: Not applicable
EpiCast: Epidemiological Forecasting
USD: US dollar
HHS: The U.S. Department of Health and Human Services

## Footnotes

1 This work is focused on influenza pandemics, and the analysis and writing presented here occurred primarily before the start of the coronavirus diseases 2019 (COVID-19) pandemic in 2020. As such, several references to pandemics and large-scale closing of schools do not take into account what had occurred during that coronavirus-associated pandemic.
