# Supplementary material for: Cost effectiveness of preemptive school closures to mitigate pandemic influenza outbreaks of differing severity in the United States
Source: BMC Public Health. 2024 Jan 17;24:200. doi: 10.1186/s12889-023-17469-8 (PMC10792817; doi:10.1186/s12889-023-17469-8)
Supplement: Supplementary file 1 — Additional file 1. [file 12889_2023_17469_MOESM1_ESM.docx]

**Supplemental Information: Cost Effectiveness of Preemptive School Closures to Mitigate Pandemic Influenza Outbreaks of Differing Severity in the United States**

Lori R Dauelsberg^1^, Brian Maskery^2*^, Heesoo Joo^2^,

Timothy C Germann^3^, Sara Y Del Valle^1^, Amra Uzicanin^2*^

^1^Analytics, Intelligence and Technology Division, Los Alamos National Laboratory,

PO Box 1663, Los Alamos, NM 87545 United States

^2^Division of Global Migration and Quarantine, Centers for Disease Control and Prevention,

1600 Clifton Road NE, MS H16-4, Atlanta, GA 30329 United States

^3^Theoretical Division, Los Alamos National Laboratory

**Table of Contents**

Contents

[1. Parameter Values 2](#_Toc152768975)

[Adjustment parameters for estimating the cost of school closures 3](#_Toc152768976)

[Potential for telecommuting in the event of school closures 4](#_Toc152768977)

[Available data from past school closures 4](#_Toc152768978)

[Other Modeling Assumptions 8](#_Toc152768979)

[2. Model equations 8](#_Toc152768980)

[3. Supplementary results: tables 11](#_Toc152768981)

[4. Supplementary results: Figures 23](#_Toc152768982)

[References 57](#_Toc152768983)

# Parameter Values

The sources of the baseline parameters for the national model for the 1957-like pandemic are given in Table 1 of the manuscript. The values for both the base case scenario as well as the uncertainty ranges to account for potential variation in the parameter values are shown in this table. The outputs from the Germann et al. analysis are summarized in Table S1 by scenario [1]. In the absence of intervention, an estimated 67.6 million pandemic influenza cases would be expected. The number of cases averted in Table S1 represent the difference between the estimate without intervention and the expected number of cases with each intervention scenario from the model. This table also summarizes the lower bound number of cases averted from the Germann et al. model.

Table S1. Summary of National Model Outcomes from Germann et al. [1] for a pandemic influenza scenario based on the 1957 influenza A(H2N2) pandemic

| **Closure Weeks** | **Trigger for Dismissal** | **Geographic Scale** | **Total Cases Averted** | **Schools Closed (%)** | **Delay to Peak (Days)** | **Lower Bound Cases Averted (Fig 1 from Germann et al.) ^a^** |
| --- | --- | --- | --- | --- | --- | --- |
| 2 | 5% | Community | 6,602,267 | 28.6 | 10 | 4,000,000 |
| 4 | 5% | Community | 8,147,318 | 28.4 | 10 | 6,000,000 |
| 8 | 5% | Community | 10,283,901 | 28.6 | 8 | 6,200,000 |
| 12 | 5% | Community | 11,130,126 | 28.4 | 10 | 6,200,000 |
| 2 | 10% | Community | 14,473,651 | 67.5 | 26 | 10,000,000 |
| 4 | 10% | Community | 19,744,595 | 66.7 | 25 | 13,000,000 |
| 8 | 10% | Community | 27,133,582 | 65.8 | 21 | 19,000,000 |
| 12 | 10% | Community | 30,541,887 | 59.3 | 19 | 21,000,000 |
| 2 | 20% | Community | 21,199,174 | 85.6 | 53 | 11,000,000 |
| 4 | 20% | Community | 33,616,090 | 80 | 69 | 20,000,000 |
| 8 | 20% | Community | 43,883,016 | 70.3 | 58 | 31,000,000 |
| 12 | 20% | Community | 48,260,651 | 47.8 | 42 | 36,000,000 |
| 2 | 5% | County | 5,187,996 | 91.8 | 27 | 3,500,000 |
| 4 | 5% | County | 10,527,088 | 92 | 42 | 7,000,000 |
| 8 | 5% | County | 22,508,176 | 89.9 | 65 | 17,000,000 |
| 12 | 5% | County | 36,304,459 | 82.4 | 73 | 29,000,000 |
| 2 | 10% | County | 4,869,025 | 99.8 | 31 | 3,000,000 |
| 4 | 10% | County | 11,897,976 | 99.6 | 50 | 8,000,000 |
| 8 | 10% | County | 30,844,738 | 97.6 | 67 | 21,000,000 |
| 12 | 10% | County | 46,923,270 | 82.1 | 79 | 37,000,000 |
| 2 | 20% | County | 4,116,346 | 100 | 33 | 3,000,000 |
| 4 | 20% | County | 11,561,027 | 100 | 55 | 6,000,000 |
| 8 | 20% | County | 33,303,723 | 99.2 | 68 | 24,000,000 |
| 12 | 20% | County | 49,838,768 | 73.8 | 79 | 40,000,000 |
| 2 | 5% | Multi-county | 2,608,405 | 99.9 | 24 | 2,000,000 |
| 4 | 5% | Multi-county | 6,188,982 | 99.9 | 44 | 3,500,000 |
| 8 | 5% | Multi-county | 19,278,082 | 99.7 | 67 | 12,000,000 |
| 12 | 5% | Multi-county | 41,814,043 | 95.8 | 82 | 31,000,000 |
| 2 | 10% | Multi-county | 2,272,025 | 100 | 26 | 1,500,000 |
| 4 | 10% | Multi-county | 4,504,475 | 100 | 43 | 3,000,000 |
| 8 | 10% | Multi-county | 19,092,846 | 100 | 69 | 11,000,000 |
| 12 | 10% | Multi-county | 42,208,642 | 97.4 | 82 | 29,000,000 |
| 2 | 20% | Multi-county | 1,911,707 | 100 | 23 | 1,500,000 |
| 4 | 20% | Multi-county | 3,849,895 | 100 | 39 | 2,500,000 |
| 8 | 20% | Multi-county | 18,669,271 | 100 | 67 | 9,000,000 |
| 12 | 20% | Multi-county | 39,830,994 | 98.6 | 80 | 28,000,000 |

^a^ The range of effectiveness estimates considered in the sensitivity analysis is from the Lower Bound Cases Averted column and Total Cases Averted Column. The Total Cases Averted column was used as the base case estimate.

## Adjustment parameters for estimating the cost of school closures

Prior to the COVID-19 pandemic, the fraction of US school children who were home-schooled had been estimated at 3.3% [2]. In addition, among 90,400 public schools, 19,000 (21.1%) offered courses entirely online during the 2015-2016 school year. Among these schools, 11.4% offered all or most courses online. Another 29.6% offered at least some come courses online, while the remaining 58.9% offered only one or a few courses online [3]. These statistics suggest that schools would be able to continue to offer some instruction during an unplanned closure in response to an influenza outbreak. As such, we estimated that about 5.6% of students would be unaffected by school closures since they are either home-schooled or attend schools for which most courses are available online. Another 6% of students would be able to access some courses online prior to the COVID-19 pandemic.

During the COVID-19 pandemic, about 93% of households with school-age children had participated in distance learning by September 2020 [4]. This represents an upper bound estimate of the number of students who may be able to access distance learning in the event of a future influenza pandemic. However, this may be an over-estimate for schools to implement closures for shorter-term closures of between two and twelve weeks for pandemic influenza. A lower bound estimate (10%) is based on data reported prior to the COVID-19 pandemic as summarized above. The future potential for distance learning to be implemented will likely fall between these bounds as some schools will maintain capacity for distance learning, but many schools may be unable to transition immediately from in-person learning to distance learning. In addition, some studies have shown a decline in student achievement as a result of the COVID-19 pandemic, which may be associated with the replacement of in-person schooling with distance learning [5]. To account for this uncertainty, we used the midpoint of the two previously reported fractions as the most likely estimate and considered a range between the lower and upper bounds for the uncertainty analysis.

## Potential for telecommuting in the event of school closures

As of 2017, only 7% of employers made telecommuting available to most employees. However, the practice was more common among larger firms; 12% of firms with 500 or more employees offered telecommuting options to most of their employees. An estimated 3% of employees worked from home at least half of the time, while the entire work-at-home employee population was 10 to 15 times greater than the number of employees who telecommuted at least half-time [6]. This would suggest that between 30% and 45% of working adults may have access to telework on a part-time basis. The Bureau of Labor Statistics reported that 23.7% of employed persons who worked on an average day had worked at home (compared to 81.7% who worked at a workplace). However, the fraction of people working at home only worked an average 3 hours a day compared to 8 hours for those working at a workplace [7]. In addition, the sum of these percentages exceeded 100%, indicating that some persons worked at an office and at home. We note that these estimates may have changed as a result of the COVID-19 pandemic so future studies may need to take these changes into consideration.

## Available data from past school closures

Several studies have examined outcomes for past school closures for both influenza outbreaks and in response to weather-related issues such as hurricanes or flooding. These outcomes are summarized in Table S2 below. In general, the percentages of adults missing work appear to be less than may be suggested based on considering telework alone. In general, the fraction of adults who missed work due to a hurricane or flooding was 28% to 46% including 15% to 30% of households that lost wages due to the closure [8, 9]. However, even for the closure in Mississippi in response to a hurricane in which 46% of households reported missing work, about 45% of these households reported only missing 1-2 days of work during the 4-day closure. Thus, the number of workdays lost due to closure would be less than the full time period of the closure.

In comparison to the weather-related closures, only 14% to 24% of households reported missing work and between 2% and 10% lost pay during closures in response to influenza outbreaks. It is possible that more individuals would miss work because of weather-related issues in addition to those missing work because of the school closure. However, it is also possible that households were more affected by closures in the school districts in which the weather-related closures occurred. For the nationwide survey conducted with parents who experienced closures during 2009 H1N1 pandemic, about 20% reported missing work and 10% reported losing pay.

The durations of closures evaluated in these analyses (between 4 and 11 days) were generally shorter than the scenarios evaluated in the Germann et al. analysis [1]. In 3 of the surveys, parents were asked if they believed it would be a problem if schools were closed for one month at some point in the future. Between 7% and 36% of households believed that such a closure would be a major or moderate problem [10-15]. Thus, the majority believed a 1-month closure would be less than a moderate problem.

Despite the lack of data on how households would be affected by a longer-term closure or on the fraction of households in which at least one parent may be able to telecommute, we applied the US national-level reported rate of 20% missing work [11] as the baseline estimate. To be conservative, we assumed that parents would miss work for the full period of closures. However, it is possible that some parents would still be able to work part-time even if they missed some time at work.

**Table S2**. Summary of published studies on the impact of unplanned school closures on households

| **Reference** | **North Carolina county, US [14]** | **Southeast KY [10]** | **US nationwide survey [11]** | **Japan [13]** | **Denver (metro), CO [15]** | **Western KY [12]** |
| --- | --- | --- | --- | --- | --- | --- |
| Timeline for closure | Nov 2-Nov 12, 2006 (11 days) | February 4-7, 2008 | Any time since the opening of school in late summer or fall 2009 | Experienced class suspension at least once between May 2009 and the time of enrolment. | Jan 29 to Feb 5, 2013 (6 days) | Jan 29 to Feb 1, 2013 (4 days) |
| Reason for closure | Influenza B virus outbreak | Influenza-like illness (ILI) absenteeism | 2009 Influenza A (H1N1) pandemic | 2009 H1N1 influenza pandemic | ILI absenteeism | ILI absenteeism |
| District size | 18,421 | 7,300 | NA | NA | 20 | 524 |
| Perceived difficulty for a 1-month future closure (moderate or major problem) | NA | NA | NA | NA | 23% | 7% |
| Response rate | 12.60% | 43.3% | 40.40% | NA | 31% | 33% |
| Fraction with difficulty | NA | NA | 23% | NA | 20.00% | 25.00% |
| Childcare | NA | NA | NA | NA | NA | NA |
| Non-working adult in home | NA | 39.8% | NA | NA | 31% | 41% |
| Adult working outside home | NA | 29.1% | NA | NA | 14% | 20% |
| Child old enough to care for him/herself | NA | NA | 10% | 28.50% | 17% | 8% |
| Other adult | NA | NA | 20% | NA | 9% | 20% |
| Older sibling | NA | NA | NA | NA | 11% | 7% |
| Adult working from home | NA | 14.9% | NA | NA | 29% | 4% |
| Adults who missed work | 18% | 29.1% | 20% | 16% | 14.3% | 18% |
| Adults who lost pay | 1.80% | NA | 10% | NA | NA | 10% |

NA- Not available in the published study

## Other Modeling Assumptions

For the cases averted, the cost of treatment is given by age range from a recent published analysis (see Table 1) [16]. Treatment costs were defined to include direct medical costs incurred from visiting healthcare providers and estimated out-of-pocket costs for non-medically attended cases. The age-specific cost of illness was estimated by subdividing populations by age and by whether individuals in each age group had at least one high-risk condition, which leads to higher estimates for the cost of both inpatient and outpatient treatment. The average cost per case was assumed to increase with age as summarized in Table 1 based on a previous analysis [16]. The ≥65 years group has the highest average treatment cost per case estimate ($9,293) and a hospitalization rate that was estimated to be more than 26 times greater than that for any other age group ($127 through $476). To account for uncertainty in treatment and indirect cost per case estimates, these parameters were varied by +/-25% in the sensitivity analysis.

Although it is possible for multiple children in a family to be sick at the same time, we assume that only one child will be sick at a time. An unreported analysis of the data from Germann et al.’s model showed that with the average infectious period (and subsequent number of days home from work or school) of 1.6 days, there is almost no overlap of multiple sick children on the same days. The average, for example, from the A (2009-like) model, is 0.02, i.e., only 2% of cases are children in the same family sick on the same days.

The base case scenario assumes that teachers and other school staff will not be paid during the closures. This may not be a likely case since it is probable that unions will insist teachers are paid. However, in the longer school closures scenarios, the lost school time may be made up during the summer or with other options such as online classrooms. The cost impact of these options is explored in the uncertainty analysis section.

# Model equations

The cost of cases averted is defined as:

$$\boldsymbol{Cost} \boldsymbol{of} \boldsymbol{cases} \boldsymbol{averted}=\boldsymbol{treatment} \boldsymbol{costs} \boldsymbol{of} \boldsymbol{all} \boldsymbol{cases} \boldsymbol{averted} +\boldsymbol{productivity} \boldsymbol{loss} \boldsymbol{of} \boldsymbol{ill} \boldsymbol{workers}+\boldsymbol{productivity} \boldsymbol{loss} \boldsymbol{of} \boldsymbol{parents} \boldsymbol{of} \boldsymbol{ill} \boldsymbol{children}$$

where

$$\boldsymbol{Treatment cost of all cases averted}= \sum_{age} [cases {averted}_{age}\times(fraction of cases who sought {treatment}_{age}\times cost to {treat}_{age}+fraction of cases who did not seek {treatment}_{age}\times did not seek treatment cost)]$$

$$\boldsymbol{Productivity loss of ill workers}=\left[ \left( number of cases averted \left( ages 19-64 \right)\times fraction of adult population working \right) + \left( number of cases averted \left( ages 65+ \right)\times fraction of working senior population \right) \right] \times average daily wages\times\left( 1 + nonwage benefits rate \right) \times number of days out sick \left( worker \right)$$

$$\boldsymbol{Productivity loss of parents of ill children}=number of cases averted for children 12 and under \times fraction of working adult population \times average daily wages\times(1+nonwage benefits rate)\times number of days out sick \left( children \right)$$

In the presence of school closures, the cost of school closures is defined as:

$$\boldsymbol{Cost of school closures=Productivity loss of school staff+ Productivity loss of parents}$$

where

$$\boldsymbol{Productivity loss of school staff}=number of students affected \times staff per student ratio \times staff wages \times(1+nonwage benefits rate) \times number of days out of school\times(1 - fraction of distance learning)$$

$$\boldsymbol{Productivity loss of parents}=\left[ \left( number of children affected by closure\div average number of children per household \right) \right]\times average daily wages\times\left( 1+ nonwage benefits rate \right)\times number of days out of school\times fraction of households with children whoes parents missed work$$

# Supplementary results: tables

**Table S3.** Summary of school closure costs and averted costs of illness by scenario: National model (1957-like pandemic)

| **Closure Weeks** | **Dismissal trigger** | **Geographic scale** | **Cases averted (millions)** | **Deaths averted (thou-sands)** | **Life-years gained (thou-sands)** | **Schools closed (%)** | **Delay to peak (days)** | **Cost of school closures (billion USD)** | **Averted cost of illness (billion USD)** | **Net cost (billion USD)** | **Net cost per case averted (1000 USD per case averted)** | **Net cost per death averted (million USD per death averted)** | **Net cost per discounted life-year gained (1000 USD per discounted life-year gained)** |
| --- | --- | --- | --- | --- | --- | --- | --- | --- | --- | --- | --- | --- | --- |
| 2 | 5% | Community | 6.6 | 15.5 | 207 | 28.6 | 10 | $11.8 | $6.9 | $4.9 | $0.7 | $0.32 | $24 |
| 2 | 5% | County | 5.2 | 16.7 | 212 | 91.8 | 27 | $30.4 | $6.6 | $23.9 | $4.6 | $1.4 | $112 |
| 2 | 5% | Multi-county | 2.6 | 8.4 | 106 | 99.9 | 24 | $32.8 | $3.3 | $29.5 | $11.3 | $3.5 | $278 |
| 2 | 10% | Community | 14.5 | 41.9 | 542 | 67.5 | 26 | $24.6 | $17.1 | $7.5 | $0.5 | $0.18 | $14 |
| 2 | 10% | County | 4.9 | 16.2 | 205 | 99.8 | 31 | $32.7 | $6.3 | $26.4 | $5.4 | $1.6 | $129 |
| 2 | 10% | Multi-county | 2.3 | 7.1 | 90 | 100 | 26 | $32.8 | $2.8 | $30.0 | $13.2 | $4.2 | $331 |
| 2 | 20% | Community | 21.2 | 67.7 | 864 | 85.6 | 53 | $29.7 | $26.7 | $3.0 | $0.1 | $0.0 | $3 |
| 2 | 20% | County | 4.1 | 13.6 | 172 | 100 | 33 | $32.8 | $5.3 | $27.5 | $6.7 | $2.0 | $160 |
| 2 | 20% | Multi-county | 1.9 | 5.8 | 75 | 100 | 23 | $32.8 | $2.3 | $30.5 | $15.9 | $5.2 | $408 |
| 4 | 5% | Community | 8.1 | 19.0 | 255 | 28.4 | 10 | $23.5 | $8.5 | $15.0 | $1.8 | $0.8 | $59 |
| 4 | 5% | County | 10.5 | 35.1 | 444 | 92 | 42 | $60.9 | $13.6 | $47.3 | $4.5 | $1.3 | $106 |
| 4 | 5% | Multi-county | 6.2 | 20.9 | 264 | 99.9 | 44 | $65.5 | $8.1 | $57.4 | $9.3 | $2.7 | $217 |
| 4 | 10% | Community | 19.7 | 56.6 | 733 | 66.7 | 25 | $48.7 | $23.2 | $25.5 | $1.3 | $0.5 | $35 |
| 4 | 10% | County | 11.9 | 40.8 | 515 | 99.6 | 50 | $65.4 | $15.7 | $49.7 | $4.2 | $1.2 | $96 |
| 4 | 10% | Multi-county | 4.5 | 15.0 | 190 | 100 | 43 | $65.6 | $5.8 | $59.7 | $13.3 | $4.0 | $314 |
| 4 | 20% | Community | 33.6 | 105.4 | 1,350 | 80 | 69 | $56.1 | $41.8 | $14.2 | $0.4 | $0.1 | $11 |
| 4 | 20% | County | 11.6 | 39.5 | 499 | 100 | 55 | $65.6 | $15.2 | $50.3 | $4.4 | $1.3 | $101 |
| 4 | 20% | Multi-county | 3.8 | 12.9 | 163 | 100 | 39 | $65.6 | $5.0 | $60.6 | $15.7 | $4.7 | $372 |
| 8 | 5% | Community | 10.3 | 23.2 | 314 | 28.6 | 8 | $47.2 | $10.5 | $36.8 | $3.6 | $1.6 | $117 |
| 8 | 5% | County | 22.5 | 75.0 | 953 | 89.9 | 65 | $119.3 | $29.1 | $90.2 | $4.0 | $1.2 | $95 |
| 8 | 5% | Multi-county | 19.3 | 65.8 | 833 | 99.7 | 67 | $130.7 | $25.4 | $105.4 | $5.5 | $1.6 | $126 |
| 8 | 10% | Community | 27.1 | 75.6 | 984 | 65.8 | 21 | $96.4 | $31.3 | $65.0 | $2.4 | $0.9 | $66 |
| 8 | 10% | County | 30.8 | 104.6 | 1,326 | 97.6 | 67 | $128.4 | $40.4 | $87.9 | $2.9 | $0.8 | $66 |
| 8 | 10% | Multi-county | 19.1 | 64.5 | 817 | 100 | 69 | $131.1 | $24.9 | $106.2 | $5.6 | $1.6 | $130 |
| 8 | 20% | Community | 43.9 | 134.4 | 1,729 | 70.3 | 58 | $99.9 | $53.8 | $46.1 | $1.1 | $0.34 | $27 |
| 8 | 20% | County | 33.3 | 113.5 | 1,438 | 99.2 | 68 | $130.2 | $43.8 | $86.4 | $2.6 | $0.8 | $60 |
| 8 | 20% | Multi-county | 18.7 | 63.4 | 803 | 100 | 67 | $131.1 | $24.5 | $106.7 | $5.7 | $1.7 | $133 |
| 12 | 5% | Community | 11.1 | 24.9 | 336 | 28.4 | 10 | $70.5 | $11.3 | $59.2 | $5.3 | $2.4 | $176 |
| 12 | 5% | County | 36.3 | 118.8 | 1,515 | 82.4 | 73 | $164.8 | $46.5 | $118.3 | $3.3 | $1.0 | $78 |
| 12 | 5% | Multi-county | 41.8 | 138.7 | 1,766 | 95.8 | 82 | $189.0 | $54.0 | $135.0 | $3.2 | $1.0 | $76 |
| 12 | 10% | Community | 30.5 | 83.6 | 1,093 | 59.3 | 19 | $131.9 | $34.9 | $97.0 | $3.2 | $1.2 | $89 |
| 12 | 10% | County | 46.9 | 155.6 | 1,981 | 82.1 | 79 | $163.7 | $60.6 | $103.1 | $2.2 | $0.66 | $52 |
| 12 | 10% | Multi-county | 42.2 | 140.0 | 1,782 | 97.4 | 82 | $191.9 | $54.5 | $137.4 | $3.3 | $1.0 | $77 |
| 12 | 20% | Community | 48.3 | 146.0 | 1,883 | 47.8 | 42 | $105.1 | $58.7 | $46.4 | $1.0 | $0.32 | $25 |
| 12 | 20% | County | 49.8 | 165.2 | 2,105 | 73.8 | 79 | $148.2 | $64.4 | $83.9 | $1.7 | $0.51 | $40 |
| 12 | 20% | Multi-county | 39.8 | 132.5 | 1,686 | 98.6 | 80 | $194.0 | $51.6 | $142.4 | $3.6 | $1.1 | $84 |

**Table S4.** Average cost per discounted life-year gained relative to no intervention (1000 USD): National model

|  | **2 weeks** | **4 weeks** | **8 weeks** | **12 weeks** |
| --- | --- | --- | --- | --- |
| **Community** | $14 | $35 | $66 | $89 |
| **County** | $129 | $96 | $66 | $52 |
| **Multi-county** | $331 | $314 | $130 | $77 |

**Table S5**. Incremental cost per discounted life-year gained relative calculated by geographical scale relative to duration of closure, e.g., 2 weeks vs. 0 weeks, 4 weeks vs. 2 weeks, 8 weeks vs. 4 weeks, and 12 weeks vs. 8 weeks (1000 USD): National model

|  | **2 weeks vs. 0 weeks** | **4 weeks vs. 2 weeks** | **8 weeks vs. 4 weeks** | **12 weeks vs. 8 weeks** |
| --- | --- | --- | --- | --- |
| **Community** | $14 | $94 | $157 | $295 |
| **County** | $129 | $75 | $47 | $23 |
| **Multi-county** | $331 | $298 | $74 | $32 |

**Table S6.** Summary of school closure costs and averted costs of illness by scenario: Chicago B2 model (1957 like pandemic)

| **Closure Weeks** | **Dismissal trigger** | **Geographic scale** | **Cases averted (thousands)** | **Deaths averted** | **Life-years gained** | **Schools closed (%)** | **Delay to Peak (Days)** | **Cost of School Closures (million USD)** | **Averted cost of illness (million USD)** | **Net cost (million USD)** | **Net cost per case averted (1000 USD per case averted)** | **Net cost per death averted (million USD per death averted)** | **Net cost per discounted life-year gained (1000 USD per discounted life-year gained)** |
| --- | --- | --- | --- | --- | --- | --- | --- | --- | --- | --- | --- | --- | --- |
| 2 | 5% | Community | 331 | 734 | 10,190 | 50 | 5 | $637 | $329 | $309 | $1 | $0 | $30 |
| 2 | 5% | Regional | 4 | 22 | 268 | 100 | 17 | $1,084 | $7 | $1,077 | $269 | $48 | $4,019 |
| 2 | 10% | Community | 391 | 1,011 | 13,625 | 83 | 15 | $969 | $425 | $544 | $1 | $1 | $40 |
| 2 | 10% | Regional | 3 | 4 | 70 | 100 | 16 | $1,084 | $2 | $1,082 | $410 | $251 | $15,459 |
| 2 | 20% | Community | 305 | 865 | 11,449 | 93 | 45 | $1,053 | $350 | $703 | $2 | $1 | $61 |
| 2 | 20% | Regional | 5 | 20 | 264 | 100 | 15 | $1,084 | $7 | $1,077 | $218 | $53 | $4,073 |
| 4 | 5% | Community | 429 | 949 | 13,183 | 51 | 5 | $1,280 | $426 | $855 | $2 | $1 | $65 |
| 4 | 5% | Regional | 12 | 37 | 497 | 100 | 35 | $2,168 | $15 | $2,153 | $187 | $58 | $4,336 |
| 4 | 10% | Community | 591 | 1,517 | 20,475 | 82 | 10 | $1,923 | $638 | $1,285 | $2 | $1 | $63 |
| 4 | 10% | Regional | 10 | 32 | 428 | 100 | 33 | $2,168 | $12 | $2,156 | $222 | $67 | $5,036 |
| 4 | 20% | Community | 584 | 1,614 | 21,520 | 92 | 70 | $2,096 | $661 | $1,435 | $2 | $1 | $67 |
| 4 | 20% | Regional | 7 | 23 | 295 | 100 | 29 | $2,168 | $9 | $2,159 | $325 | $95 | $7,331 |
| 8 | 5% | Community | 548 | 1,183 | 16,518 | 50 | 5 | $2,536 | $536 | $2,000 | $4 | $2 | $121 |
| 8 | 5% | Regional | 112 | 347 | 4,550 | 100 | 65 | $4,336 | $137 | $4,199 | $37 | $12 | $923 |
| 8 | 10% | Community | 999 | 2,528 | 34,276 | 82 | 8 | $3,855 | $1,070 | $2,784 | $3 | $1 | $81 |
| 8 | 10% | Regional | 97 | 306 | 4,010 | 100 | 64 | $4,336 | $120 | $4,216 | $43 | $14 | $1,051 |
| 8 | 20% | Community | 1,182 | 3,219 | 43,135 | 90 | 86 | $4,102 | $1,325 | $2,777 | $2 | $1 | $64 |
| 8 | 20% | Regional | 70 | 219 | 2,870 | 100 | 59 | $4,336 | $86 | $4,250 | $61 | $19 | $1,481 |
| 12 | 5% | Community | 593 | 1,286 | 17,942 | 50 | 4 | $3,794 | $581 | $3,213 | $5 | $2 | $179 |
| 12 | 5% | Regional | 745 | 2,145 | 28,517 | 100 | 89 | $6,504 | $867 | $5,637 | $8 | $3 | $198 |
| 12 | 10% | Community | 1,237 | 3,087 | 42,013 | 81 | 10 | $5,728 | $1,314 | $4,413 | $4 | $1 | $105 |
| 12 | 10% | Regional | 652 | 1,891 | 25,084 | 100 | 87 | $6,504 | $762 | $5,742 | $9 | $3 | $229 |
| 12 | 20% | Community | 1,627 | 4,380 | 58,932 | 85 | 18 | $5,842 | $1,810 | $4,031 | $2 | $1 | $68 |
| 12 | 20% | Regional | 506 | 1,474 | 19,537 | 100 | 84 | $6,504 | $593 | $5,911 | $12 | $4 | $303 |

**Table S7.** Average cost per discounted life-year gained relative to no intervention (1000 USD): Chicago B2 model (1957 like pandemic)

|  | **2 weeks** | **4 weeks** | **8 weeks** | **12 weeks** |
| --- | --- | --- | --- | --- |
| **Community** | 40 | 63 | 81 | 105 |
| **Regional** | 15,459 | 5,036 | 1,051 | 229 |

**Table S8**. Incremental cost per discounted life-year gained relative calculated by geographical scale relative to duration of closure, e.g., 2 weeks vs. 0 weeks, 4 weeks vs. 2 weeks, 8 weeks vs. 4 weeks, and 12 weeks vs. 8 weeks (1000 USD): Chicago B2 model (1957 like pandemic)

|  | **2 weeks vs. 0 weeks** | **4 weeks vs. 2 weeks** | **8 weeks vs. 4 weeks** | **12 weeks vs. 8 weeks** |
| --- | --- | --- | --- | --- |
| **Community** | 40 | 108 | 109 | 211 |
| **Regional** | 15,459 | 2,999 | 575 | 72 |

**Table S9.** Summary of school closure costs and averted costs of illness by scenario: Chicago B1 model (1968 like pandemic)

| **Closure Weeks** | **Dismissal trigger** | **Geographic scale** | **Cases averted (thousands)** | **Deaths averted** | **Life-years gained** | **Schools closed (%)** | **Delay to Peak (Days)** | **Cost of School Closures (million USD)** | **Averted cost of illness (million USD)** | **Net cost (million USD)** | **Net cost per case averted (1000 USD per case averted)** | **Net cost per death averted (million USD per death averted)** | **Net cost per discounted life-year gained (1000 USD per discounted life-year gained)** |
| --- | --- | --- | --- | --- | --- | --- | --- | --- | --- | --- | --- | --- | --- |
| 2 | 5% | Community | 172 | 78 | 1,195 | 23 | 4 | $321 | $96 | $225 | $1.31 | $3 | $188 |
| 2 | 5% | Regional | 28 | 19 | 276 | 100 | 18 | $1,084 | $17 | $1,067 | $38.67 | $55 | $3,861 |
| 2 | 10% | Community | 400 | 218 | 3,245 | 61 | 13 | $751 | $234 | $517 | $1.29 | $2 | $159 |
| 2 | 10% | Regional | 18 | 13 | 185 | 100 | 16 | $1,084 | $12 | $1,072 | $58.68 | $81 | $5,801 |
| 2 | 20% | Community | 568 | 335 | 4,941 | 83 | 46 | $967 | $339 | $628 | $1.11 | $2 | $127 |
| 2 | 20% | Regional | 20 | 15 | 205 | 100 | 16 | $1,084 | $12 | $1,071 | $54.91 | $73 | $5,225 |
| 4 | 5% | Community | 209 | 97 | 1,477 | 23 | 3 | $640 | $118 | $522 | $2.50 | $5 | $354 |
| 4 | 5% | Regional | 83 | 55 | 791 | 100 | 35 | $2,168 | $51 | $2,116 | $25.46 | $38 | $2,677 |
| 4 | 10% | Community | 544 | 293 | 4,382 | 60 | 11 | $1,488 | $317 | $1,171 | $2.15 | $4 | $267 |
| 4 | 10% | Regional | 78 | 52 | 749 | 100 | 34 | $2,168 | $49 | $2,119 | $27.11 | $40 | $2,830 |
| 4 | 20% | Community | 857 | 499 | 7,398 | 80 | 59 | $1,868 | $509 | $1,359 | $1.59 | $3 | $184 |
| 4 | 20% | Regional | 47 | 32 | 447 | 100 | 29 | $2,168 | $29 | $2,139 | $45.72 | $68 | $4,785 |
| 8 | 5% | Community | 245 | 111 | 1,713 | 23 | 2 | $1,268 | $137 | $1,131 | $4.61 | $10 | $660 |
| 8 | 5% | Regional | 600 | 368 | 5,410 | 100 | 64 | $4,336 | $363 | $3,973 | $6.62 | $11 | $734 |
| 8 | 10% | Community | 724 | 384 | 5,780 | 60 | 9 | $2,960 | $420 | $2,540 | $3.51 | $7 | $439 |
| 8 | 10% | Regional | 460 | 283 | 4,153 | 100 | 61 | $4,336 | $279 | $4,057 | $8.83 | $14 | $977 |
| 8 | 20% | Community | 1,156 | 670 | 9,979 | 73 | 34 | $3,447 | $686 | $2,761 | $2.39 | $4 | $277 |
| 8 | 20% | Regional | 372 | 229 | 3,347 | 100 | 58 | $4,336 | $226 | $4,110 | $11.05 | $18 | $1,228 |
| 12 | 5% | Community | 261 | 118 | 1,823 | 23 | 3 | $1,897 | $146 | $1,751 | $6.70 | $15 | $960 |
| 12 | 5% | Regional | 1,252 | 771 | 11,416 | 100 | 77 | $6,504 | $757 | $5,746 | $4.59 | $7 | $503 |
| 12 | 10% | Community | 803 | 425 | 6,396 | 54 | 9 | $4,047 | $465 | $3,581 | $4.46 | $8 | $560 |
| 12 | 10% | Regional | 1,117 | 684 | 10,120 | 100 | 76 | $6,504 | $675 | $5,829 | $5.22 | $9 | $576 |
| 12 | 20% | Community | 1,293 | 745 | 11,133 | 57 | 20 | $4,080 | $766 | $3,313 | $2.56 | $4 | $298 |
| 12 | 20% | Regional | 1,036 | 637 | 9,410 | 100 | 73 | $6,504 | $627 | $5,877 | $5.67 | $9 | $625 |

**Table S10.** Average cost per discounted life-year gained relative to no intervention (1000 USD): Chicago B1 model (1968 like pandemic)

|  | **2 weeks** | **4 weeks** | **8 weeks** | **12 weeks** |
| --- | --- | --- | --- | --- |
| **Community** | 159 | 267 | 439 | 560 |
| **Regional** | 5,801 | 2,830 | 977 | 576 |

**Table S11**. Incremental cost per discounted life-year gained relative calculated by geographical scale relative to duration of closure, e.g., 2 weeks vs. 0 weeks, 4 weeks vs. 2 weeks, 8 weeks vs. 4 weeks, and 12 weeks vs. 8 weeks (1000 USD): Chicago B1 model (1968 like pandemic)

|  | **2 weeks vs. 0 weeks** | **4 weeks vs. 2 weeks** | **8 weeks vs. 4 weeks** | **12 weeks vs. 8 weeks** |
| --- | --- | --- | --- | --- |
| **Community** | 159 | 575 | 979 | 1,690 |
| **Regional** | 5,801 | 1,856 | 569 | 297 |

**Table S12.** Summary of school closure costs and averted costs of illness by scenario: Chicago A model (2009 like pandemic)

| **Closure Weeks** | **Dismissal trigger** | **Geographic scale** | **Cases averted (thousands)** | **Deaths averted** | **Life-years gained** | **Schools closed (%)** | **Delay to Peak (Days)** | **Cost of School Closures (million USD)** | **Averted cost of illness (million USD)** | **Net cost (million USD)** | **Net cost per case averted (1000 USD per case averted)** | **Net cost per death averted (million USD per death averted)** | **Net cost per discounted life-year gained (1000 USD per discounted life-year gained)** |
| --- | --- | --- | --- | --- | --- | --- | --- | --- | --- | --- | --- | --- | --- |
| 2 | 5% | Community | 152 | 120 | 1,756 | 16 | 6 | $223 | $96 | $127 | $0.83 | $1 | $72 |
| 2 | 5% | Regional | 95 | 100 | 1,379 | 100 | 20 | $1,084 | $64 | $1,020 | $10.79 | $10 | $740 |
| 2 | 10% | Community | 483 | 456 | 6,470 | 47 | 20 | $591 | $317 | $274 | $0.57 | $1 | $42 |
| 2 | 10% | Regional | 88 | 92 | 1,279 | 100 | 17 | $1,084 | $59 | $1,025 | $11.69 | $11 | $801 |
| 2 | 20% | Community | 862 | 875 | 12,282 | 60 | 43 | $729 | $576 | $153 | $0.18 | $0 | $12 |
| 2 | 20% | Regional | 109 | 115 | 1,599 | 100 | 23 | $1,084 | $74 | $1,010 | $9.25 | $9 | $632 |
| 4 | 5% | Community | 178 | 142 | 2,089 | 16 | 5 | $449 | $113 | $336 | $1.89 | $2 | $161 |
| 4 | 5% | Regional | 300 | 319 | 4,419 | 100 | 37 | $2,168 | $203 | $1,965 | $6.55 | $6 | $445 |
| 4 | 10% | Community | 572 | 534 | 7,592 | 46 | 17 | $1,171 | $375 | $796 | $1.39 | $1 | $105 |
| 4 | 10% | Regional | 296 | 314 | 4,348 | 100 | 38 | $2,168 | $200 | $1,968 | $6.65 | $6 | $453 |
| 4 | 20% | Community | 1,070 | 1,084 | 15,237 | 53 | 44 | $1,280 | $715 | $565 | $0.53 | $1 | $37 |
| 4 | 20% | Regional | 217 | 233 | 3,220 | 100 | 33 | $2,168 | $147 | $2,020 | $9.30 | $9 | $628 |
| 8 | 5% | Community | 191 | 151 | 2,221 | 16 | 5 | $885 | $121 | $764 | $4.00 | $5 | $344 |
| 8 | 5% | Regional | 878 | 928 | 12,923 | 100 | 53 | $4,336 | $593 | $3,742 | $4.26 | $4 | $290 |
| 8 | 10% | Community | 644 | 595 | 8,488 | 44 | 14 | $2,219 | $421 | $1,798 | $2.79 | $3 | $212 |
| 8 | 10% | Regional | 753 | 794 | 11,052 | 100 | 51 | $4,336 | $509 | $3,827 | $5.08 | $5 | $346 |
| 8 | 20% | Community | 1,153 | 1,163 | 16,370 | 42 | 41 | $2,037 | $770 | $1,267 | $1.10 | $1 | $77 |
| 8 | 20% | Regional | 667 | 702 | 9,773 | 100 | 49 | $4,336 | $450 | $3,885 | $5.82 | $6 | $398 |
| 12 | 5% | Community | 201 | 163 | 2,371 | 14 | 6 | $1,178 | $128 | $1,050 | $5.24 | $6 | $443 |
| 12 | 5% | Regional | 1,327 | 1,391 | 19,470 | 100 | 61 | $6,504 | $896 | $5,608 | $4.23 | $4 | $288 |
| 12 | 10% | Community | 681 | 631 | 8,998 | 31 | 13 | $2,391 | $445 | $1,946 | $2.86 | $3 | $216 |
| 12 | 10% | Regional | 1,195 | 1,254 | 17,530 | 100 | 59 | $6,504 | $806 | $5,697 | $4.77 | $5 | $325 |
| 12 | 20% | Community | 1,195 | 1,206 | 16,976 | 25 | 38 | $1,857 | $798 | $1,059 | $0.89 | $1 | $62 |
| 12 | 20% | Regional | 1,156 | 1,215 | 16,974 | 100 | 60 | $6,504 | $781 | $5,723 | $4.95 | $5 | $337 |

**Table S13.** Average cost per discounted life-year gained relative to no intervention (1000 USD): Chicago A model (2009 like pandemic)

|  | **2 weeks** | **4 weeks** | **8 weeks** | **12 weeks** |
| --- | --- | --- | --- | --- |
| **Community** | 42 | 105 | 212 | 216 |
| **Regional** | 801 | 453 | 346 | 325 |

**Table S14**. Incremental cost per discounted life-year gained relative calculated by geographical scale relative to duration of closure, e.g., 2 weeks vs. 0 weeks, 4 weeks vs. 2 weeks, 8 weeks vs. 4 weeks, and 12 weeks vs. 8 weeks (1000 USD): Chicago A model (2009 like pandemic)

|  | **2 weeks vs. 0 weeks** | **4 weeks vs. 2 weeks** | **8 weeks vs. 4 weeks** | **12 weeks vs. 8 weeks** |
| --- | --- | --- | --- | --- |
| **Community** | 42 | 465 | 1,119 | 290 |
| **Regional** | 801 | 307 | 277 | 289 |

**Table S15**. Distribution of cases averted by age group for each scenario, National model (1957-like pandemic)

| **Closure weeks** | **Dismissal trigger** | **Geographic scale** | **0-4** | **5-18** | **19-29** | **30-64** | **65+** |
| --- | --- | --- | --- | --- | --- | --- | --- |
| 2 | 5% | Community | 12% | 48% | 14% | 21% | 5% |
|  |  | County | 10% | 43% | 17% | 24% | 7% |
|  |  | Multi-county | 10% | 44% | 16% | 23% | 7% |
|  | 10% | Community | 10% | 45% | 16% | 23% | 6% |
|  |  | County | 9% | 42% | 17% | 24% | 7% |
|  |  | Multi-county | 10% | 44% | 16% | 23% | 7% |
|  | 20% | Community | 9% | 42% | 17% | 24% | 7% |
|  |  | County | 10% | 42% | 17% | 24% | 7% |
|  |  | Multi-county | 10% | 44% | 16% | 23% | 7% |
| 4 | 5% | Community | 12% | 48% | 14% | 21% | 5% |
|  |  | County | 9% | 42% | 17% | 25% | 7% |
|  |  | Multi-county | 9% | 41% | 17% | 25% | 7% |
|  | 10% | Community | 10% | 45% | 16% | 23% | 6% |
|  |  | County | 9% | 41% | 17% | 25% | 8% |
|  |  | Multi-county | 10% | 41% | 17% | 24% | 7% |
|  | 20% | Community | 9% | 42% | 17% | 25% | 7% |
|  |  | County | 9% | 41% | 17% | 25% | 8% |
|  |  | Multi-county | 10% | 42% | 17% | 24% | 7% |
| 8 | 5% | Community | 12% | 49% | 14% | 21% | 5% |
|  |  | County | 9% | 41% | 17% | 25% | 7% |
|  |  | Multi-county | 9% | 40% | 18% | 25% | 8% |
|  | 10% | Community | 10% | 45% | 16% | 23% | 6% |
|  |  | County | 9% | 40% | 18% | 26% | 8% |
|  |  | Multi-county | 9% | 41% | 17% | 25% | 7% |
|  | 20% | Community | 9% | 43% | 17% | 25% | 7% |
|  |  | County | 9% | 40% | 18% | 26% | 8% |
|  |  | Multi-county | 9% | 40% | 18% | 25% | 8% |
| 12 | 5% | Community | 12% | 49% | 14% | 21% | 5% |
|  |  | County | 9% | 41% | 17% | 25% | 7% |
|  |  | Multi-county | 9% | 40% | 18% | 26% | 7% |
|  | 10% | Community | 10% | 45% | 16% | 23% | 6% |
|  |  | County | 9% | 40% | 18% | 26% | 7% |
|  |  | Multi-county | 9% | 40% | 18% | 26% | 7% |
|  | 20% | Community | 9% | 43% | 17% | 25% | 7% |
|  |  | County | 9% | 40% | 18% | 26% | 7% |
|  |  | Multi-county | 9% | 40% | 18% | 26% | 7% |

**Table S16**. Distribution of cases averted by age group for each scenario, Chicago B2 model (1957 like pandemic)

| **Closure Weeks** | **Dismissal trigger** | **Geographic scale** | **0-4** | **5-18** | **19-29** | **30-64** | **65+** |
| --- | --- | --- | --- | --- | --- | --- | --- |
| 2 | 5% | Community | 13% | 48% | 14% | 21% | 4% |
|  |  | Regional | 6% | 32% | 23% | 26% | 13% |
|  | 10% | Community | 12% | 46% | 15% | 22% | 5% |
|  |  | Regional | 4% | 41% | 12% | 40% | 2% |
|  | 20% | Community | 12% | 45% | 15% | 22% | 6% |
|  |  | Regional | 9% | 24% | 28% | 31% | 9% |
| 4 | 5% | Community | 13% | 48% | 14% | 21% | 4% |
|  |  | Regional | 11% | 34% | 16% | 32% | 7% |
|  | 10% | Community | 12% | 46% | 15% | 22% | 5% |
|  |  | Regional | 6% | 35% | 24% | 28% | 7% |
|  | 20% | Community | 12% | 44% | 16% | 23% | 6% |
|  |  | Regional | 6% | 40% | 16% | 30% | 7% |
| 8 | 5% | Community | 13% | 49% | 14% | 21% | 4% |
|  |  | Regional | 13% | 41% | 16% | 24% | 7% |
|  | 10% | Community | 11% | 46% | 15% | 22% | 5% |
|  |  | Regional | 13% | 40% | 16% | 24% | 7% |
|  | 20% | Community | 11% | 44% | 16% | 23% | 6% |
|  |  | Regional | 12% | 40% | 17% | 24% | 7% |
| 12 | 5% | Community | 12% | 49% | 14% | 21% | 4% |
|  |  | Regional | 12% | 42% | 16% | 24% | 6% |
|  | 10% | Community | 11% | 46% | 15% | 22% | 5% |
|  |  | Regional | 12% | 42% | 16% | 23% | 6% |
|  | 20% | Community | 11% | 44% | 16% | 24% | 6% |
|  |  | Regional | 12% | 42% | 16% | 23% | 6% |

**Table S17**. Distribution of cases averted by age group for each scenario, Chicago B1 model (1968 like pandemic)

| **Closure Weeks** | **Dismissal trigger** | **Geographic scale** | **0-4** | **5-18** | **19-29** | **30-64** | **65+** |
| --- | --- | --- | --- | --- | --- | --- | --- |
| 2 | 5% | Community | 13% | 50% | 14% | 20% | 4% |
|  |  | Regional | 9% | 42% | 17% | 25% | 7% |
|  | 10% | Community | 11% | 47% | 15% | 22% | 5% |
|  |  | Regional | 11% | 42% | 15% | 25% | 7% |
|  | 20% | Community | 10% | 44% | 16% | 24% | 5% |
|  |  | Regional | 10% | 41% | 19% | 24% | 8% |
| 4 | 5% | Community | 13% | 50% | 14% | 20% | 4% |
|  |  | Regional | 11% | 42% | 16% | 24% | 6% |
|  | 10% | Community | 11% | 46% | 15% | 23% | 5% |
|  |  | Regional | 11% | 42% | 16% | 24% | 7% |
|  | 20% | Community | 10% | 44% | 16% | 24% | 5% |
|  |  | Regional | 12% | 43% | 15% | 24% | 7% |
| 8 | 5% | Community | 12% | 49% | 14% | 21% | 4% |
|  |  | Regional | 11% | 42% | 17% | 24% | 6% |
|  | 10% | Community | 11% | 46% | 15% | 23% | 5% |
|  |  | Regional | 11% | 43% | 16% | 24% | 6% |
|  | 20% | Community | 10% | 44% | 17% | 24% | 5% |
|  |  | Regional | 11% | 43% | 16% | 24% | 6% |
| 12 | 5% | Community | 12% | 49% | 14% | 20% | 4% |
|  |  | Regional | 10% | 42% | 17% | 25% | 6% |
|  | 10% | Community | 11% | 46% | 15% | 23% | 5% |
|  |  | Regional | 10% | 42% | 17% | 25% | 6% |
|  | 20% | Community | 10% | 44% | 17% | 24% | 5% |
|  |  | Regional | 10% | 42% | 17% | 25% | 6% |

**Table S18**. Distribution of cases averted by age group for each scenario, Chicago A model (2009 like pandemic)

| **Closure Weeks** | **Dismissal trigger** | **Geographic scale** | **0-4** | **5-18** | **19-29** | **30-64** | **65+** |
| --- | --- | --- | --- | --- | --- | --- | --- |
| 2 | 5% | Community | 13% | 50% | 13% | 20% | 4% |
|  |  | Regional | 11% | 44% | 16% | 24% | 5% |
|  | 10% | Community | 11% | 46% | 15% | 23% | 4% |
|  |  | Regional | 11% | 44% | 15% | 24% | 5% |
|  | 20% | Community | 10% | 44% | 16% | 24% | 5% |
|  |  | Regional | 11% | 43% | 16% | 24% | 5% |
| 4 | 5% | Community | 13% | 49% | 14% | 21% | 4% |
|  |  | Regional | 11% | 43% | 16% | 24% | 5% |
|  | 10% | Community | 11% | 46% | 15% | 23% | 4% |
|  |  | Regional | 11% | 43% | 16% | 24% | 5% |
|  | 20% | Community | 10% | 44% | 17% | 24% | 5% |
|  |  | Regional | 11% | 43% | 16% | 24% | 5% |
| 8 | 5% | Community | 13% | 49% | 14% | 21% | 4% |
|  |  | Regional | 10% | 43% | 17% | 25% | 5% |
|  | 10% | Community | 11% | 46% | 15% | 23% | 4% |
|  |  | Regional | 10% | 43% | 17% | 25% | 5% |
|  | 20% | Community | 10% | 44% | 17% | 24% | 5% |
|  |  | Regional | 11% | 43% | 17% | 24% | 5% |
| 12 | 5% | Community | 13% | 49% | 14% | 21% | 4% |
|  |  | Regional | 10% | 43% | 17% | 25% | 5% |
|  | 10% | Community | 11% | 46% | 15% | 23% | 4% |
|  |  | Regional | 10% | 43% | 17% | 25% | 5% |
|  | 20% | Community | 10% | 44% | 17% | 24% | 5% |
|  |  | Regional | 10% | 43% | 17% | 25% | 5% |

# Supplementary results: Figures

**Figure S1a.** Effect of increasing the duration (2, 4, 8, 12 weeks) of school closures (while keeping the dismissal decision and geographic scale constant (within each scenario subgroup as separated by the vertical lines) on cases averted, delay to peak, and net cost

**National-level** strategy against an influenza pandemic similar to the **1957 A (H2N2) pandemic**

**Chicago strategy** against an influenza pandemic similar to the **1957 A(H2N2) pandemic**

**Chicago strategy** against an influenza pandemic similar to the **1968 A(H3N2) pandemic**

**Chicago strategy** against an influenza pandemic similar to the **2009 A(H1N1) pandemic**

**Figure S1b.** Effect of increasing the dismissal trigger decision (5%, 10%, 20%) for school closures (while keeping the duration and geographic scale constant within each scenario subgroup as separated by the vertical lines) on cases averted, delay to peak, and net cost

**National model** against an influenza pandemic similar to the **1957 A(H2N2) pandemic**

**Chicago B2 model** against an influenza pandemic similar to the **1957 A(H2N2) pandemic**

**Chicago B1 model** against an influenza pandemic similar to the **1968 A(H3N2) pandemic**

**Chicago A model** against an influenza pandemic similar to the **2009 A(H1N1) pandemic**

**Figure S1c.** Effect of changing the school geographic scale (community, county, multi-county for the National model and community or Regional for the Chicago models) from community to multi-county (while keeping the dismissal decision and duration constant within each scenario subgroup as separated by the vertical lines) on cases averted, delay to peak, and net cost

**National model** against an influenza pandemic similar to the **1957 A(H2N2) pandemic**

Note: “Com” stands for community, and “Multi” stands for “multi-county”

**Chicago B2 model** against an influenza pandemic similar to the **1957 A(H2N2) pandemic**

**Chicago B1 model** against an influenza pandemic similar to the **1968 A(H3N2) pandemic**

**Chicago** **A model** against an influenza pandemic similar to the **2009 A(H1N1) pandemic**

**Figure S2a**. Impact of trigger* for school dismissal decision during an influenza pandemic on net costs and number of life-years gained for **community-level** school closures, 1957-like pandemic, National model

*The trigger is linked to assumptions about the sensitivity of the surveillance system used to detect infected schoolchildren based on the diagnostic ratio. The diagnostic ratio is based on the fraction of symptomatic children infected with influenza who would be detected. With a lower diagnostic ratio/trigger, it takes longer for schools to begin to close because infected school children are detected less readily (i.e., for a diagnostic ratio of 5%, only 5 out of 100 children with symptomatic influenza infections would be detected). This paper focused on an assumed diagnostic ratio of 10% and provided estimates for 5% and 20% diagnostic ratios in the sensitivity analyses and supplemental information.

**Figure S2b**. Impact of trigger* for school dismissal decision during an influenza pandemic on net costs and number of life-years gained for **county-level** school closures, 1957-like pandemic, National model

*The trigger is linked to assumptions about the sensitivity of the surveillance system used to detect infected schoolchildren based on the diagnostic ratio. The diagnostic ratio is based on the fraction of symptomatic children infected with influenza who would be detected. With a lower diagnostic ratio/trigger, it takes longer for schools to begin to close because infected school children are detected less readily (i.e., for a diagnostic ratio of 5%, only 5 out of 100 children with symptomatic influenza infections would be detected). This paper focused on an assumed diagnostic ratio of 10% and provided estimates for 5% and 20% diagnostic ratios in the sensitivity analyses and supplemental information.

**Figure S2c**. Impact of trigger* for school dismissal decision during an influenza pandemic on net costs and number of life-years gained for **multi-county–level** school closures, 1957-like pandemic, National model

*The trigger is linked to assumptions about the sensitivity of the surveillance system used to detect infected schoolchildren based on the diagnostic ratio. The diagnostic ratio is based on the fraction of symptomatic children infected with influenza who would be detected. With a lower diagnostic ratio/trigger, it takes longer for schools to begin to close because infected school children are detected less readily (i.e., for a diagnostic ratio of 5%, only 5 out of 100 children with symptomatic influenza infections would be detected). This paper focused on an assumed diagnostic ratio of 10% and provided estimates for 5% and 20% diagnostic ratios in the sensitivity analyses and supplemental information.

**Figure S3a**. Impact of trigger* for school dismissal decision during an influenza pandemic on net costs and number of life-years gained for **community-level** school closures for Chicago model

**Model B2** (1957-like pandemic)

**Model B1** (1968-like pandemic)

**Model A** (2009-like pandemic)

*The trigger is linked to assumptions about the sensitivity of the surveillance system used to detect infected schoolchildren based on the diagnostic ratio. The diagnostic ratio is based on the fraction of symptomatic children infected with influenza who would be detected. With a lower diagnostic ratio/trigger, it takes longer for schools to begin to close because infected school children are detected less readily (i.e., for a diagnostic ratio of 5%, only 5 out of 100 children with symptomatic influenza infections would be detected). This paper focused on an assumed diagnostic ratio of 10% and provided estimates for 5% and 20% diagnostic ratios in the sensitivity analyses and supplemental information.

**Figure S3b**. Impact of trigger* for school dismissal decision during an influenza pandemic on net costs and number of life-years gained for **regional-level** school closures for Chicago model

**Model B2** (1957-like pandemic)

**Model B1** (1968 like pandemic)

**Model A** (2009 like pandemic)

*The trigger is linked to assumptions about the sensitivity of the surveillance system used to detect infected schoolchildren based on the diagnostic ratio. The diagnostic ratio is based on the fraction of symptomatic children infected with influenza who would be detected. With a lower diagnostic ratio/trigger, it takes longer for schools to begin to close because infected school children are detected less readily (i.e., for a diagnostic ratio of 5%, only 5 out of 100 children with symptomatic influenza infections would be detected). This paper focused on an assumed diagnostic ratio of 10% and provided estimates for 5% and 20% diagnostic ratios in the sensitivity analyses and supplemental information.

**Figure S4**. Scatterplot of the net cost per case averted and days to peak for school dismissal decisions during a 1957-like pandemic, National model

**Chicago B2 model** (1957-like pandemic)

**Chicago B1 model** (1968-like pandemic)

**Chicago A model** (2009-like pandemic)

Note: Mult = Multi-county, Comm = Community, Coun = County, Regi = Regional

**Figure S5a.** Effect of increasing the **school dismissal** **duration** (while keeping constant the school dismissal geographic scale) on cases averted, delay to peak, and net cost

**Chicago** **B2 model** against an influenza pandemic similar to the **1957 A(H2N2) pandemic**

**Chicago** **B1 model** against an influenza pandemic similar to the **1968 A(H3N2) pandemic**

**Chicago** **A model** against an influenza pandemic similar to the **2009 A(H1N1) pandemic**

Note: School dismissal trigger is 10% for Figure S5a. The trigger is linked to assumptions about the sensitivity of the surveillance system used to detect infected schoolchildren based on the diagnostic ratio. The diagnostic ratio is based on the fraction of symptomatic children infected with influenza who would be detected. With a lower diagnostic ratio/trigger, it takes longer for schools to begin to close because infected school children are detected less readily (i.e., for a diagnostic ratio of 5%, only 5 out of 100 children with symptomatic influenza infections would be detected). This paper focused on an assumed diagnostic ratio of 10% and provided estimates for 5% and 20% diagnostic ratios in the sensitivity analyses and supplemental information.

**Figure S5b.** Effect of increasing **the school dismissal** **geographic scale** (while keeping the school dismissal duration constant) on cases averted, delay to peak, and net cost

**Chicago** **B2 model** against an influenza pandemic similar to the **1957 A(H2N2) pandemic**

**Chicago B1 model** against an influenza pandemic similar to the **1968 A(H3N2) pandemic**

**Chicago A model** against an influenza pandemic similar to the **2009 A(H1N1) pandemic**

Note: School dismissal trigger is 10% for Figure A 5b. The trigger is linked to assumptions about the sensitivity of the surveillance system used to detect infected schoolchildren based on the diagnostic ratio. The diagnostic ratio is based on the fraction of symptomatic children infected with influenza who would be detected. With a lower diagnostic ratio/trigger, it takes longer for schools to begin to close because infected school children are detected less readily (i.e., for a diagnostic ratio of 5%, only 5 out of 100 children with symptomatic influenza infections would be detected). This paper focused on an assumed diagnostic ratio of 10% and provided estimates for 5% and 20% diagnostic ratios in the sensitivity analyses and supplemental information.

**Figure S6**. **Net cost** (billion USD) and **number of life-years gained** for community and regional geographic scale school dismissal during an influenza pandemic (Net cost per discounted life-year gained, thousand USD reported as data labels)

**Chicago B2 model** against an influenza pandemic similar to the **1957 A(H2N2) pandemic**

**Chicago B1 model** against an influenza pandemic similar to the **1968 A(H3N2) pandemic**

**Chicago A model** against an influenza pandemic similar to the **2009 A(H1N1) pandemic**

Notes: School dismissal trigger is 10% for Figure A 5c. The school dismissal duration increases from 2 weeks to 4 weeks to 8 weeks to 12 weeks for each series by school dismissal geographic scale. The net costs and life-years gained increase with the durations of closures for each geographic scale.The trigger is linked to assumptions about the sensitivity of the surveillance system used to detect infected schoolchildren based on the diagnostic ratio. The diagnostic ratio is based on the fraction of symptomatic children infected with influenza who would be detected. With a lower diagnostic ratio/trigger, it takes longer for schools to begin to close because infected school children are detected less readily (i.e., for a diagnostic ratio of 5%, only 5 out of 100 children with symptomatic influenza infections would be detected). This paper focused on an assumed diagnostic ratio of 10% and provided estimates for 5% and 20% diagnostic ratios in the sensitivity analyses and supplemental information.

**Figure S7**. **Net cost** (billion USD) of school closure and **number of life-years gained** for 1957-, 1968-, and 2009-like pandemic models in Chicago (Net cost per discounted life-year gained, thousand USD reported as data labels)

Geographic scale: **Community**

Geographic scale: **Regional**

**Figure S8**. One-way sensitivity analyses of the **net cost of school closure** per relative parameter to the 4-week county-level closure with a 10% dismissal decision diagnosis ratio assumption* at base case values

**Chicago** **B2 model** against an influenza pandemic similar to the **1957 A(H2N2) pandemic**

**Chicago B1 model** against an influenza pandemic similar to the **1968 A(H3N2) pandemic**

**Chicago A model** against an influenza pandemic similar to the **2009 A(H1N1) pandemic**

The trigger is linked to assumptions about the sensitivity of the surveillance system used to detect infected schoolchildren based on the diagnostic ratio. The diagnostic ratio is based on the fraction of symptomatic children infected with influenza who would be detected. With a lower diagnostic ratio/trigger, it takes longer for schools to begin to close because infected school children are detected less readily (i.e., for a diagnostic ratio of 5%, only 5 out of 100 children with symptomatic influenza infections would be detected). This paper focused on an assumed diagnostic ratio of 10% and provided estimates for 5% and 20% diagnostic ratios in the sensitivity analyses and supplemental information.

**Figure S9**. One-way sensitivity analyses of the **net cost per case averted** by school closures during an influenza pandemic relative to the 4-week county-level closure with a 10% diagnostic ratio assumption* at base case values

**Chicago** **B2 model** against an influenza pandemic similar to the **1957 A(H2N2) pandemic**

**Chicago B1** **model** against an influenza pandemic similar to the **1968 A(H3N2) pandemic**

**Chicago** **A** **model** against an influenza pandemic similar to the **2009 A(H1N1) pandemic**

The trigger is linked to assumptions about the sensitivity of the surveillance system used to detect infected schoolchildren based on the diagnostic ratio. The diagnostic ratio is based on the fraction of symptomatic children infected with influenza who would be detected. With a lower diagnostic ratio/trigger, it takes longer for schools to begin to close because infected school children are detected less readily (i.e., for a diagnostic ratio of 5%, only 5 out of 100 children with symptomatic influenza infections would be detected). This paper focused on an assumed diagnostic ratio of 10% and provided estimates for 5% and 20% diagnostic ratios in the sensitivity analyses and supplemental information.

**Figure S10**. One-way sensitivity analyses of the **net cost per death averted** by school closures during an influenza pandemic relative to the 4-week county-level closure with a 10% diagnosis ratio assumption* at base case values

**Chicago** **B2 model** against an influenza pandemic similar to the **1957 A(H2N2) pandemic**

**Chicago** **B1 model** against an influenza pandemic similar to the **1968 A(H3N2) pandemic**

**Chicago** **A model** against an influenza pandemic similar to the **2009 A(H1N1) pandemic**

The trigger is linked to assumptions about the sensitivity of the surveillance system used to detect infected schoolchildren based on the diagnostic ratio. The diagnostic ratio is based on the fraction of symptomatic children infected with influenza who would be detected. With a lower diagnostic ratio/trigger, it takes longer for schools to begin to close because infected school children are detected less readily (i.e., for a diagnostic ratio of 5%, only 5 out of 100 children with symptomatic influenza infections would be detected). This paper focused on an assumed diagnostic ratio of 10% and provided estimates for 5% and 20% diagnostic ratios in the sensitivity analyses and supplemental information.

**Figure S11**. One-way sensitivity analyses of the **net cost per discounted life-year gained** by school closures during an influenza pandemic relative to the 4-week county-level closure with a 10% diagnosis ratio assumption* at base case values

**Chicago** **B2 model** against an influenza pandemic similar to the **1957 A(H2N2) pandemic**

**Chicago** **B1 model** against an influenza pandemic similar to the **1968 A(H3N2) pandemic**

**Chicago A model** strategy against an influenza pandemic similar to the **2009 A(H1N1) pandemic**

The trigger is linked to assumptions about the sensitivity of the surveillance system used to detect infected schoolchildren based on the diagnostic ratio. The diagnostic ratio is based on the fraction of symptomatic children infected with influenza who would be detected. With a lower diagnostic ratio/trigger, it takes longer for schools to begin to close because infected school children are detected less readily (i.e., for a diagnostic ratio of 5%, only 5 out of 100 children with symptomatic influenza infections would be detected). This paper focused on an assumed diagnostic ratio of 10% and provided estimates for 5% and 20% diagnostic ratios in the sensitivity analyses and supplemental information.

# References

1. Germann TC, Gao H, Gambhir M, Plummer A, Biggerstaff M, Reed C, Uzicanin A: **School dismissal as a pandemic influenza response: When, where and for how long?** *Epidemics* 2019, **28**:100348.

2. McQuiggan M, Megra M, Grady S: **Parent and family involvement in education: Results from the National Household Education Surveys program of 2016: First look. Washington, DC: U.S. Department of Education. NCES 2017-102. Retrieved September 26, 2017 from** [**https://nces.ed.gov/pubs2017/2017102.pdf**](https://nces.ed.gov/pubs2017/2017102.pdf). 2017.

3. Taie S, Goldring R: **Characteristics of Public Elementary and Secondary Schools in the United States: Results From the 2015–16 National Teacher and Principal Survey First Look (NCES 2017-071). U.S. Department of Education. Washington, DC: National Center for Education Statistics. Retrieved 3/31/2020 from** [**https://nces.ed.gov/pubsearch/pubsinfo.asp?pubid=2017071**](https://nces.ed.gov/pubsearch/pubsinfo.asp?pubid=2017071)**.** 2017.

4. Mcelrath K: **Schooling During the COVID-19 Pandemic. United Census Bureau. (August 26, 2020)**[**https://www.census.gov/library/stories/2020/08/schooling-during-the-covid-19-pandemic.html**](https://www.census.gov/library/stories/2020/08/schooling-during-the-covid-19-pandemic.html)**. Accessed June 24, 2021.** 2020.

5. Donnelly R, Patrinos HA: **Learning loss during Covid‑19: An early systematic review**. *Prospects* 2021, **Nov 10**:1-9.

6. Global Workplace Analytics, Flexjobs: **State of Telecommuting in the U.S. Employee Workforce.** [**https://www.flexjobs.com/2017-State-of-Telecommuting-US/#formstart**](https://www.flexjobs.com/2017-State-of-Telecommuting-US/#formstart)**. Accessed September 27, 2019.** 2017.

7. Bureau of Labor Statistics: **Bureau of Labor Statistics. American Time Use Survey - 2018 Results** [**https://www.bls.gov/news.release/pdf/atus.pdf**](https://www.bls.gov/news.release/pdf/atus.pdf)**. Accessed September 27, 2019**. 2019.

8. Zheteyeva Y, Rainey J, Gao H, Jacobson E, Adhikari B, Shi J, et al.: **Unintended costs and consequences of school closures implemented in preparation for Hurricane Isaac in Harrison County School District, Mississippi, August-September 2012.** *PLoS ONE* 2017, **12**(11):e0184326.

9. Tsai V, Khan NM, Shi J, Rainey J, Gao H, Zheteyeva Y: **Evaluation of Unintended Social and Economic Consequences of an Unplanned School Closure in Rural Illinois**. *J Sch Health* 2017, **87**(7):546-553.

10. Timperio CG, Humbaugh K, Riggs M, Thoroughman D, Barrios L, Copeland D, Waller A, Denniston M, Beavers S, 1405-1409 CBM: **Impact of Seasonal Influenza-Related School Closures on Families --- Southeastern Kentucky, February 2008**. *MMWR Recomm Rep* 2009, **58**(50):1405-1409.

11. Steelfisher GK, Blendon RJ, Bekheit MM, Liddon N, Kahn E, Schieber R, Lubell K: **Steelfisher, G. K., et al. "Parental attitudes and experiences during school dismissals related to 2009 influenza A (H1N1)-United States, 2009." Morbidity and Mortality Weekly Report59.35 (2010): 1131-1134**. *MMWR Week Rep* 2010, **59**(35):1131-1134.

12. Russell ES, Zheteyeva Y, Gao H, et al.: **Reactive school closure during increased influenza-like illness (ILI) activity in western Kentucky, 2013: a field evaluation of effect on ILI incidence and economic and social consequences for families.** . *Open Forum Infect Diseases* 2016, **3**(3):ofw113.

13. Mizumoto K, Yamamoto T, Nishiura H: **Contact behaviour of children and parental employment behaviour during school closures against the pandemic influenza A (H1N1-2009) in Japan**. *Journal of International Medical Research* 2013, **41**(3):716-724.

14. Johnson AJ, Moore ZS, Edelson PJ, Kinnane L, Davies M, Shay DK, Balish A, McCarron M, Blanton L, Finelli L *et al*: **Johnson, April J., et al. "Household responses to school closure resulting from outbreak of influenza B, North Carolina." Emerging infectious diseases 14.7 (2008): 1024**. *Emerg Inf Dis* 2008, **14**(7).

15. Epson E, Zheteyeva Y, Rainey J, et al.: **Evaluation of an unplanned school closure in a Colorado school district: implications for pandemic influenza preparedness**. *Disaster Med Public Health Prep* 2015, **9**(1):4-8.

16. Biggerstaff M, Chen J, Rolfes M, O'Halloran A, Garg S, Zhou F, Burns E, Jernigan D, Reed C: **The Economic Burden of Influenza in the United States since 2009. Options X for Control of Influenza: 28 August - September 1, 2019 in Suntec Singapore.** 2019.
